# Manipulating PP2Acα-ASK-JNK signaling to favor apoptotic over necroptotic hepatocyte fate reduces the extent of necrosis and fibrosis upon acute liver injury

**DOI:** 10.1038/s41419-022-05353-z

**Published:** 2022-11-22

**Authors:** Ke Lu, Si-Yu Shen, Ou-Yang Luo, Yue Lu, Tian-Shu Shi, Jing Wu, Qi Cheng, Hua-Jian Teng, Di Chen, Xiang Lu, Chao-Jun Li, Qing Jiang, Lei Fang, Bin Xue

**Affiliations:** 1 Core Laboratory, Sir Run Run Hospital, Nanjing Medical University, Nanjing, 211166 China; 2 State Key Laboratory of Pharmaceutical Biotechnology, Division of Sports Medicine and Adult Reconstructive Surgery, Department of Orthopedic Surgery, Nanjing Drum Tower Hospital, The Affiliated Hospital of Nanjing University Medical School, 321 Zhongshan Road, Nanjing, 210008 Jiangsu China; 3 Branch of National Clinical Research Center for Orthopedics, Sports Medicine and Rehabilitation, Nanjing, China; 4 Faculty of Pharmaceutical Sciences, Shenzhen Institute of Advanced Technology, Chinese Academy of Sciences, Shenzhen, 518055 China; 5 State Key Laboratory of Pharmaceutical Biotechnology, Nanjing University Medical School, Nanjing, 210046 China; 6 Key Laboratory of Model Animal for Disease Study of Ministry of Education, Model Animal Research Center, Nanjing University, Nanjing, 210032 China; 7 Department of Geriatrics, Sir Run Run Hospital, Nanjing Medical University, Nanjing, 211166 China; 8 Key Laboratory for Aging and Disease, Nanjing Medical University, Nanjing, 211166 China; 9 State Key Laboratory of Reproductive Medicine and China International Joint Research Center on Environment and Human Health, Center for Global Health, School of Public Health, Nanjing Medical University, Nanjing, 211166 China; 10 Collaborative Innovation Center For Cancer Personalized Medicine, Nanjing Medical University, Nanjing, China

**Keywords:** Cell death, Mechanisms of disease

## Abstract

In the widely used Carbon tetrachloride (CCl_4_)-induced acute liver injury (ALI) mouse model, hepatocytes are known to die from programmed cell death (PCD) processes including apoptosis and necroptosis. Both in vivo and in vitro experiments showed that CCl_4_ treatment could induce both apoptosis and necroptosis. Treatment of mice with the apoptosis inducer SMAC mimetic reduced necroptosis, led to less pronounced liver damage, and improved overall liver function. By LC-MS/MS, we found that PP2Acα expression was increased in ALI mice liver, and we confirmed its high expression in subacute hepatitis patients. We observed that ALI severity (including aggravated fibrogenesis) was significantly alleviated in hepatocyte-specific *PP2Acα* conditional knockout (*PP2Acα* cKO) mice. Furthermore, the relative extent of apoptosis over necroptosis was increased in the *PP2Acα* cKO ALI mice. Pursuing the idea that biasing the type of PCD towards apoptosis may reduce liver damage, we found that treatment of *PP2Acα* cKO ALI mice with the apoptosis inhibitor z-Vad-fmk increased the extent of necroptosis and caused severer damage. Mechanistically, disruption of PP2Acα prevents the dephosphorylation of pASK1(Ser967), thereby preventing the sustained activation of JNK. Inhibition of PP2Acα prevents CCl_4_-induced liver injury and fibrogenesis by disrupting ASK/JNK pathway mediated PCD signaling, ultimately improving liver function by biasing hepatocytes towards an apoptotic rather than necroptotic cell fate. Thus, targeting PP2A and/or ASK1 to favor apoptotic over necroptotic hepatocyte fate may represent an attractive therapeutic strategy for treating ALI.

## Introduction

Acute liver injury (ALI) is a major cause of acute liver failure. ALI is commonly a result of drug and alcohol abuse, exposure to toxins, hepatitis virus infection, and ischemia/reperfusion, and leads to life-threatening liver failure, chronic hepatic steatosis, and fibrosis [1]. ALI progression is characterized by excessive cell death in hepatocytes, and both apoptosis and necroptosis are known to mediate ALI-associated cell death. Clinical and animal studies with disease models including non-alcoholic steatohepatitis, chronic liver injury, and hepatocellular carcinoma have revealed that both apoptosis and necroptosis occur in liver disease progression [2, 3].

Apoptosis and necroptosis are two forms of programmed cell death. Apoptosis occurs as a result of the activation of intrinsic and extrinsic stimulation that causes release of mitochondrial cytochrome c and intracellular death factors that ultimately kill cells via activated cleaved caspase-3 [4]. In contrast, necroptosis employs receptor-interacting protein kinase-3 (RIP3) to trigger the phosphorylation and activation of an executioner protein known as mixed lineage kinase domain-like (MLKL) [5, 6]. It is now established that these two modes of cell death trigger distinct responses in various tissues, with demonstrated differences for both regeneration (*e.g*., liver, bone or skin) and systemic immune responses [3, 7, 8]. Although it is known that both apoptosis and necroptosis occur extensively in ALI livers, we are unaware of research exploring potential differential impacts of these distinct cell death processes on the progression and/or severity of liver diseases.

The protein phosphatase type 2A (PP2A) is a kind of serine/threonine phosphatase that cause phosphorylation change of key control proteins that regulate biological progresses, and has been implicated in the regulation of programmed cell death and proliferation [9]. PP2A inhibitor microcystin can cause an increase of apoptosis in tumors and normal organs [10]. As the most important catalytic subunit, PP2Acα has also been shown to function in liver regeneration post-hepatectomy: it regulates the termination of regeneration processes via the AKT/GSK3β/cyclin D1 signaling pathway [11].

Carbon tetrachloride (CCl_4_) is a cytotoxic agent to hepatocytes and can cause centrilobular necrosis and fibrosis [12]. CCl_4_ is widely used to induce liver injury in models, and is also a representative hepatotoxin to study clinical liver disease [13]. It has been shown that liver-specific deletion of PP2Acα inhibits TGF-β1/Smad to attenuate CCl_4_-induced chronic liver fibrosis, suggesting a functional impact of PP2Acα in CCl_4_-induced ALI [14]. However, it is unclear if PP2Acα’s ability to regulating cell death may somehow contribute to the progression of CCl_4_-induced ALI.

In this study, we show that the deletion of *PP2Acα* promotes apoptosis over necroptosis in hepatocytes of CCl_4_-induced ALI model mice, which alleviates fibrogenesis and results in an overall improvement of liver function. Moreover, we demonstrated that targeted manipulation of PP2Acα/pASK(ser967)/pJNK signaling can bias hepatocytes to an apoptotic cell fate to reduce the deleterious effect of liver injury.

## Results

### Both apoptosis and necroptosis occur in the livers of CCl_4_-induced ALI model mice

**Fig. 1:**
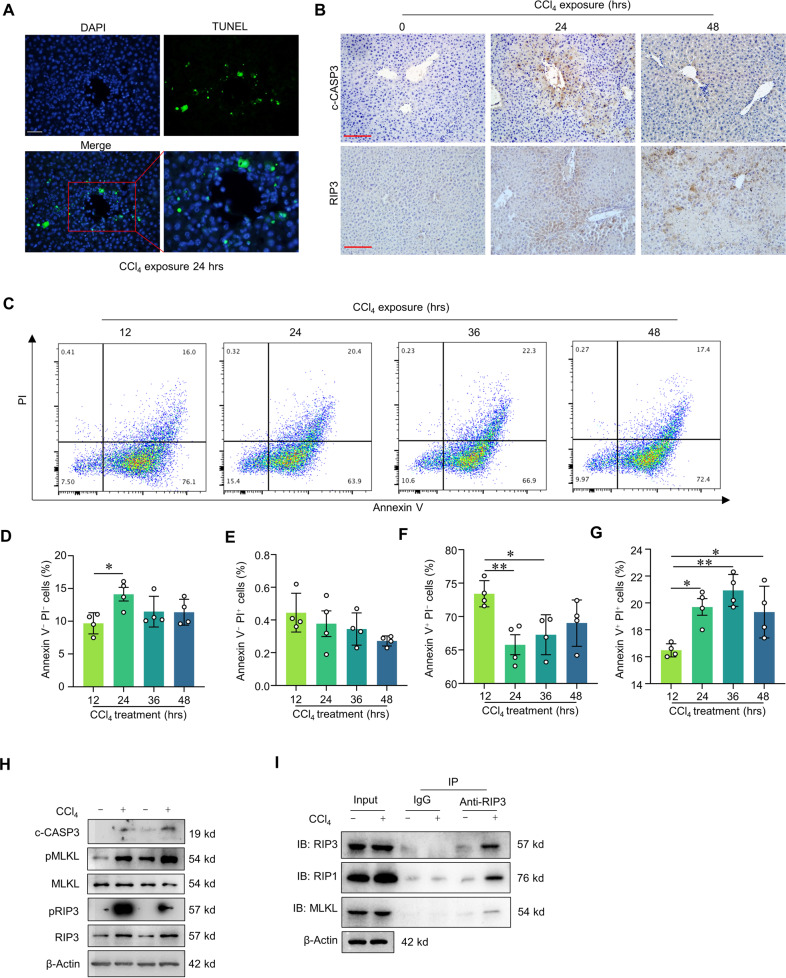
Both apoptosis and necroptosis occurred in ALI mice liver induced by CCl_4_. Representative images of TUNEL assay (**A**) and liver cleaved caspase-3 (c-CASP3) and RIP3 immunohistochemical staining (**B**) in wild-type (WT) mice with CCl_4_ induction. Scale bar: 100 μm. **C** Representative images of flow cytometry analysis based on gating for Annexin V or PI signals to assess primary hepatocytes isolated from WT mice were treated with CCl_4_ for 12, 24, 36 and 48 h. Ratio of Annexin V^−^ PI^−^ cells (**D**), PI^+^ cells (**E**) Annexin V^+^ cells (**F**) and Annexin V^+^ PI^+^ cells (**G**) in primary hepatocytes isolated from WT mice with CCl_4_ treatment for 12, 24, 36, and 48 h (*n* = 4). **H** Immunoblotting showed the liver Cleaved-Caspase-3 (c-CASP3), RIP3, pRIP3, pMLKL, and MLKL in WT mice with CCl_4_ induction. **I** Co-immunoprecipitation was conducted to detect the interactions among RIP1, RIP3, and MLKL in primary hepatocytes with PBS, TS, TSZ, and CCl_4_ treatment. **p < 0.01, one-way ANOVA followed by Tukey’s multiple comparisons test (**D**–**G**). Data are represented as mean ± SD.

To investigate cell death processes in an ALI mice model, we injected wild-type mice with CCl_4_ and it mainly damaged the central vein and portal area in the hepatic lobule. TUNEL assays showed that dead hepatocytes were induced in the livers of CCl_4_-induced ALI model mice (Fig. 1A). Immunohistochemical staining with antibodies against cleaved caspase-3 (c-CASP3) and RIP3 respectively indicated that apoptosis and necroptosis occur in mouse livers within 24 h of CCl_4_ injection (Fig. 1B). We next conducted in vitro studies to delineate any specific impacts of CCl_4_ on hepatocytes death by Annexin V/propidium iodide (PI) flow cytometry (Fig. 1C). Consistent with our detection of cell death occurring in ALI mouse livers, the isolated primary hepatocytes exhibited Annexin V^+^ and PI^+^ death signals with CCl_4_ for different time as indicated (Fig. 1D–G). The immunoblotting assay confirmed the accumulation of c-CASP3, pMLKL, MLKL pRIP3 and RIP3, also indicating the existence of apoptosis and necroptosis in ALI (Fig. 1H). To determinate necrosome formation, we performed Co-immunoprecipitation (Co-IP) to detect the interactions among RIP1, RIP3 and MLKL in primary hepatocytes isolated from the livers of CCl_4_-induced ALI model mice. As shown in Fig. 1I, CCl_4_ treatment resulted in high level of necrosome formation by RIP1-RIP3 complex and MLKL in primary hepatocytes isolated from WT mice. Together, our results demonstrated that both apoptosis and necroptosis are present in CCl_4_-induced ALI model livers and CCl_4_-treated isolated hepatocytes.

### Promoting apoptosis over necroptosis in ALI livers reduces tissue damage

**Fig. 2:**
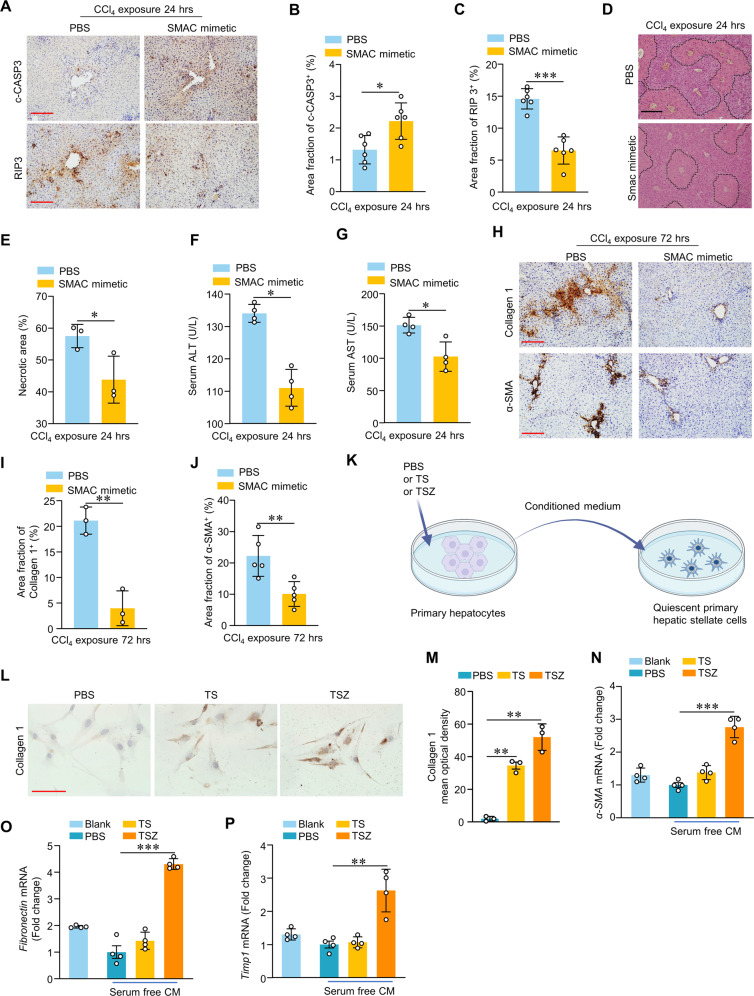
Promoting apoptosis over necroptosis reduces the extent of damage and fibrogenesis in CCl_4_-induced ALI livers. Representative images of IHC staining of liver c-CASP3 and RIP3 (**A**) (scale bar, 100 mm) and quantification of liver c-CASP3-positive area (**B**) and RIP3-positive area (**C**) in WT mice treated with CCl_4_ 24 h, followed by SMAC mimetic or PBS treatment (*n* = 6). Representative images of H&E staining of liver (**D**) (scale bar, 100 mm) and quantification of liver necrotic areas (**E**) in WT mice treated with CCl_4_ 24 h, followed by SMAC mimetic or PBS treatment (*n* = 3). Serum ALT (**F**) and AST (**G**) levels in WT mice treated as indicated. (*n* = 4). Representative images of IHC staining of liver Collagen I and α-SMA (**H**) (scale bar, 100 mm) and quantification of liver Collagen I-positive area (**I**) (*n* = 3) and α-SMA-positive area (**J**) (*n* = 5) in WT mice treated with CCl_4_ 72 h, followed by SMAC mimetic or PBS treatment. (**K**) A schematic diagram demonstrating culture of quiescent primary hepatic stellate cells (HSCs) treated with conditioned medium (CM) collected from primary hepatocytes pretreated with PBS or TS or TSZ. Representative images of IHC staining (**L**) (Scale bar: 100 μm) and quantification of Collagen I-mean optical density (**M**) (*n* = 3) in primary HSCs treated as indicated. qRT-PCR of relative fibrogenesis marker, *α-SMA* (**N**), *Fibronectin* (**O**), and *Timp1* (**P**) mRNA levels in primary HSCs treated as indicated (*n* = 4). *p < 0.05, **p < 0.01, ***p < 0.001, two-tailed Student’s unpaired *t*-test (**B**, **C**, **E**–**G**, **I**–**J**); one-way ANOVA followed by Tukey’s multiple comparisons test (**M**–**P**). Data are represented as mean ± SD.

Next, we tested whether shifting the relative frequency of apoptosis vs. necroptosis may influence the pathogenesis of ALI. We pretreated CCl_4_-induced ALI mice with SMAC Mimetic SM-164 to promote apoptosis. Compared to control mice, the liver tissues of the SM-164 treated CCl_4_-induced mice exhibited increased staining for c-CASP3 along with decreased staining for RIP3 (Fig. 2A–C), indicating relatively increased apoptosis vs. necroptosis in SM-164-treated livers. Furthermore, the SM-164 treatment resulted in decreased necrotic area (Fig. 2D, E) in reduced levels of the liver function markers ALT and AST in sera (Fig. 2F, G). SM-164 alone treatment didn’t induce liver injury in control mice (Fig. S1A–C).

Fibrogenesis in livers is known to drive the conversion of acute liver diseases into chronic disease conditions. We also found that the SM-164 treatment to promote apoptosis suppressed fibrogenesis as compared to the PBS-treated ALI model controls. Specifically, this was marked by a significant decrease in α-SMA and collagen I staining in liver tissues (Fig. 2H–J). Pursuing these observations in vitro, we treated resting hepatic stellate cells (HSCs) with media from variously treated cultured hepatocytes, including hepatocytes treated with TS+ caspase inhibitor z-VAD-fmk (TSZ) to induce necroptosis or hepatocytes treated with TNF-α+ SMAC mimetic (TS) to induce apoptosis (Fig. 2K). Compared with culture medium from necroptotic hepatocytes, the culture medium from apoptotic hepatocytes stimulated primary HSCs to express higher levels of collagen I (Fig. 2L, M). Together, these results establish that treatment with SM-164 can promote apoptosis over necroptosis in CCl_4_-induced livers and show that such promotion results in reductions in tissue damage, levels of serological markers of liver injury, and the extent of fibrosis.

### Livers from ALI mice and human subacute hepatitis patients exhibit elevated PP2Acα protein levels

**Fig. 3:**
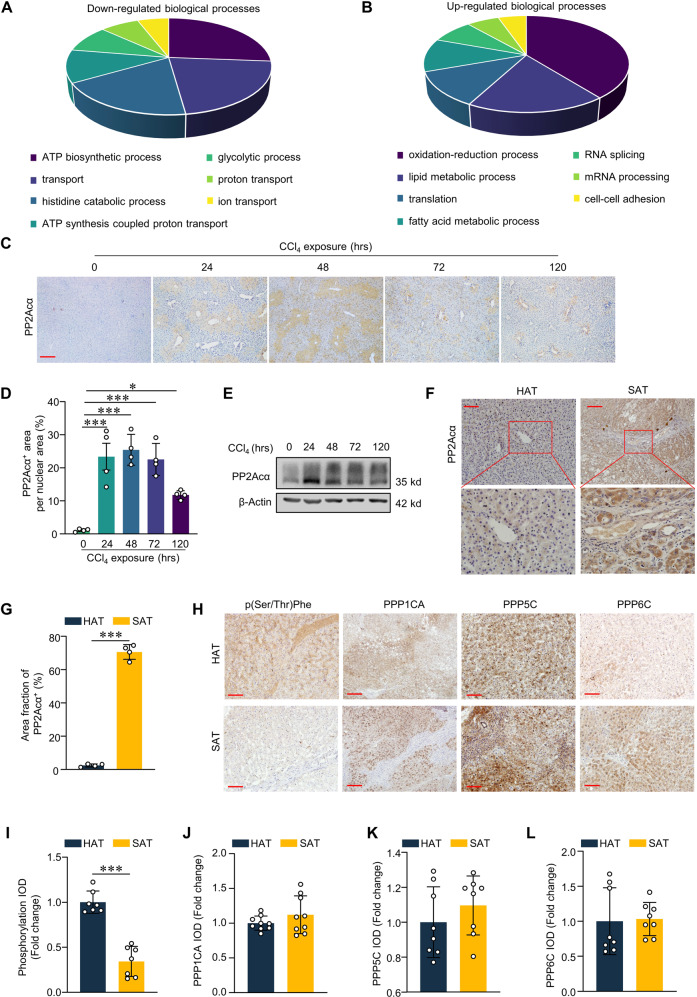
PP2Acα was involved in ALI. LC-MS/MS-based proteomic profiling. Compared with Cre^−^ mice treated with olive oil, the levels of proteins associated with down-regulated biological processes (**A**) and up-regulated biological processes (**B**) in CCl_4_-induced ALI was shown. Representative IHC images of liver PP2Acα staining (**C**) (Scale bar: 200 μm) and quantification (**D**) (*n* = 4) of PP2Acα-positive area in mice with CCl_4_ induction at the indicated time. **E** Immunoblotting of PP2Acα expression in mice liver with CCl_4_ induction at the indicated time. Representative IHC images of liver PP2Acα staining (**F**) (Scale bar: 100 μm) and quantification (**G**) (*n* = 4) of PP2Acα-positive area in hemangioma-adjacent tissues (HAT) and subacute hepatitis tissues (SAT) from patients. Representative IHC images of liver p(Ser/Thr)Phe, PPP1CA, PPP5C A and PPP6C staining (**H**) (Scale bar: 200 μm) and quantification (**I**–**L**) (*n* = 8) of integrated optical density in HAT and SAT from patients. *p < 0.05, **p < 0.01, ***p < 0.001, one-way ANOVA followed by Tukey’s multiple comparisons test (**D**); two-tailed Student’s unpaired *t*-test (**G**, **I**–**L**). Data are represented as mean ± SD.

To investigate any changes in the proteomes of the livers in the CCl_4_-induced ALI mouse model, we conducted LC-MS/MS-based proteomic profiling. Compared with control mice treated with olive oil, the levels of proteins associated with energy metabolism were significantly reduced in livers of CCl_4_-induced ALI mice, for example, reduced levels of ATP biosynthesis and proton-transporting ATP synthase proteins (Figs. 3A and S2A). This analysis also revealed that the ALI livers had increased levels of lipid metabolism proteins and proteins with oxidoreductase activity (Figs. 3B and S2B). As phosphorylation is a crucial biological progression, we explored the changes of phosphatases during CCl_4_-induced ALI. As expected, we found significant increases in the levels of the protein phosphatases PP2Acα and PP1A in CCl_4_-induced ALI mice by LC-MS/MS (Table 1). Supporting our LC-MS data, IHC with an antibody against PP2Acα showed increased signals at the central veins of livers from CCl_4_-induced ALI mice (Fig. 3C, D). Immunoblotting over a time course revealed that the protein level of PP2Acα was significantly up-regulated within 24 h of CCl_4_ injection, and this significantly elevated level remained for 120 h (Fig. 3E), again implicating PP2Acα in the pathogenesis of ALI.

**Table 1:** Protein phosphatases detected by LC-MS/MS in livers of CCl_4_-induced ALI mice.

Accession	Name	CCl4: Olive Oil	Peptides≥5
Q7TNP2	Serine/threonine-protein phosphatase 2A 65 kDa regulatory subunit A beta isoform	2.357205391	9
P63330	Serine/threonine-protein phosphatase 2A catalytic subunit alpha isoform	2.290982246	14
P49443	Protein phosphatase 1A	1.754891753	12
Q64487	Receptor-type tyrosine-protein phosphatase delta	1.468161225	5
Q922D4	Serine/threonine-protein phosphatase 6 regulatory subunit 3	1.32902348	6
Q3UM45	Protein phosphatase 1 regulatory subunit 7	1.213824868	9
Q60676	Serine/threonine -protein phosphatase 5	1.192629695	7
P29351	Tyrosine-protein phosphatase non-receptor type 6	1.17545712	6
Q9D7X3	Dual specificity protein phosphatase 3	1.036427021	7
A2A8L5	Receptor-type tyrosine-protein phosphatase F	1.004776835	6
Q6P1F6	Serine/threonine-protein phosphatase 2A 55 kDa regulatory subunit B alpha isoform	0.988638401	5
Q76MZ3	Serine/threonine-protein phosphatase 2A 65 kDa regulatory subunit A alpha isoform	0.895278394	21
P36993	Protein phosphatase 1B	0.81511724	11
Q9D358	Low molecular weight phosphotyrosine protein phosphatase	0.795439899	6
Q61074	Protein phosphatase 1G	0.793744206	6
P62715	Serine/threonine-protein phosphatase 2A catalytic subunit beta isoform	0.710960925	14

To further confirm that PP2Acα is involved in the pathogenesis of ALI, we collected liver tissues from 11 subacute hepatitis patients and 4 hemangioma patients (Table S1). We conducted immunostaining of these tissues with a variety of antibodies and found that, compared to the hemangioma-adjacent tissues, the subacute massive necrotic tissues displayed significantly increased PP2Acα accumulation (Fig. 3F, G), a higher number of inflammatory cells, atypical hepatocyte morphology, and increased fibrosis, findings collectively indicating a massive inflammatory invasion, necrosis, and fibrosis (Fig. 3F). We stained with a phospho-(Ser/Thr) Phe antibody to assess the overall extent of protein phosphorylation, and found that the subacute massive necrosis samples had obviously reduced phosphorylation levels compared to the hemangioma-adjacent control tissues (Fig. 3H, I). Note that we detected no differences in the accumulation levels of other phosphatases (*e.g*., PPP1CA, PPP5C, or PPP6C) between the necrotic and control samples from the patients (Fig. 3H, J–L), suggesting that the PP2Acα increase we observed may be specifically associated with subacute massive necrosis.

### Genetic deficiency of PP2Acα promotes apoptosis over necroptosis in ALI livers

**Fig. 4:**
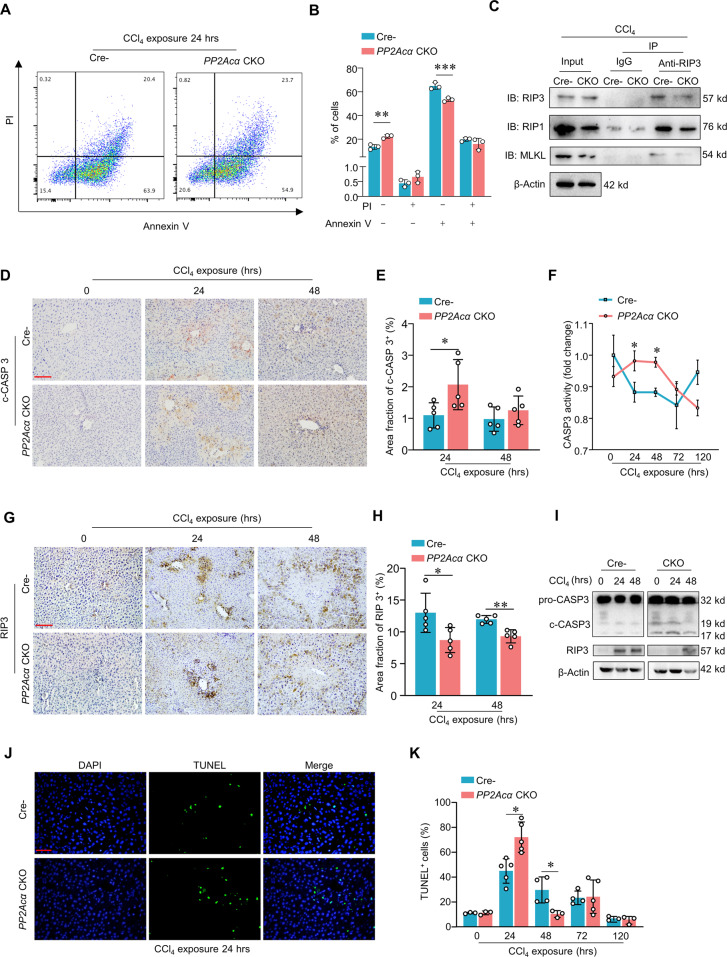
Conditional knockout of PP2Acα promotes apoptosis over necroptosis in CCl_4_-induced ALI livers. Representative images (**A**) and analysis (**B**) of flow cytometry of Annexin V and PI signals in primary hepatocytes isolated from Cre^−^ or *PP2Acα* CKO mice treated with CCl_4_ for 24 h. (*n* = 3) **C** Co-immunoprecipitation was conducted to detect the interactions among RIP1, RIP3, and MLKL in primary Cre^−^ and *PP2Acα* CKO hepatocytes with PBS, TS, TSZ, and CCl_4_ treatment. Representative images of IHC staining of liver c-CASP3 (**D**) (scale bar, 100 μm) and quantification of liver c-CASP3-positive area (**E**) in Cre^−^ and *PP2Acα* CKO mice treated with CCl_4_ 24 h and 48 h (*n* = 5). **F** Caspase-3 activity in liver tissues from Cre^−^ and *PP2Acα* CKO mice from 0 to 120 h after CCl_4_ induction (*n* = 3). Representative images of IHC staining of liver RIP3 (**G**) (scale bar, 100 μm) and quantification of liver RIP3-positive area (**H**) in Cre^−^ and *PP2Acα* CKO mice treated with CCl_4_ 24 h and 48 h (*n* = 5). **I** Immunoblotting showed the liver Pro-Caspase-3 (pro-CASP3), c-CASP3, RIP3 in Cre^−^ and *PP2Acα* CKO mice with CCl_4_ induction. Representative images (**J**) (scale bar, 100 μm) and quantification of TUNEL staining (**K**) (*n* = 3–5) of hepatocytes from Cre^−^ and *PP2Acα* CKO mice at indicated time after CCl_4_ induction. *p < 0.05, **p < 0.01, ***p < 0.001, one-way ANOVA followed by Tukey’s multiple comparisons test (**B**, **E**, **F**, **H** and **K**). Data are represented as mean ± SD.

Recall our observations that both apoptosis and necroptosis occur in CCl_4_-induced ALI livers and that these livers exhibit increased PP2Acα levels (Fig. 1 and Table S2). Thus, we wondered whether PP2Acα may regulate one or both of the PCD types we observed in ALI livers. Pursuing this, we isolated primary hepatocytes from mice that have conditional knockout of *PP2Acα* specifically in hepatocytes (*PP2Acα* cKO) (Fig. S3A, B) and treated with CCl_4_. We found that the *PP2Acα* cKO primary hepatocytes had relatively more Annexin V^−^ PI^−^ cells and less Annexin V^+^ PI^+^ cells with CCL_4_ treatment for 24 h, compared to hepatocytes isolated from control littermates (Cre^−^ mice) (Fig. 4A, B). We then performed Co-IP to determinate necrosome formation in mice with CCl_4_ treatment for 24 h. The results showed the protein level of RIP1-RIP3-MLKL complex was lower in *PP2Acα* cKO livers compared with that in Cre^−^ mice livers (Fig. 4C).

We also explored the impact of *PP2Acα* in vivo by using CCl_4_ to induce liver damage in *PP2Acα* cKO mice and measuring apoptosis and necroptosis in livers. Compared to Cre^−^ ALI model livers, the *PP2Acα* cKO livers had higher level of c-CASP3 (58% increase) (Fig. 4D, E) and increased CASP3 activity (Fig. 4F), and had reduced RIP3 levels (24% decrease) (Fig. 4G, H). Immunoblotting further confirmed the c-CASP3 elevation and reduced RIP3 accumulation in the *PP2Acα* cKO livers (Fig. 4I). Moreover, the number of apoptotic cells detected by TUNEL staining was significantly higher in *PP2Acα* cKO mice than that in control mice at 24 h post CCl_4_ induction (Fig. 4J, K). Together, these results demonstrate that genetic deficiency for *PP2Acα* shifts the type of PCD that occurs in the pathogenesis of ALI, with the absence of *PP2Acα* in hepatocytes clearly promoting apoptosis over necrosis.

### PP2Acα deficiency alleviates liver damage in ALI model mice

**Fig. 5:**
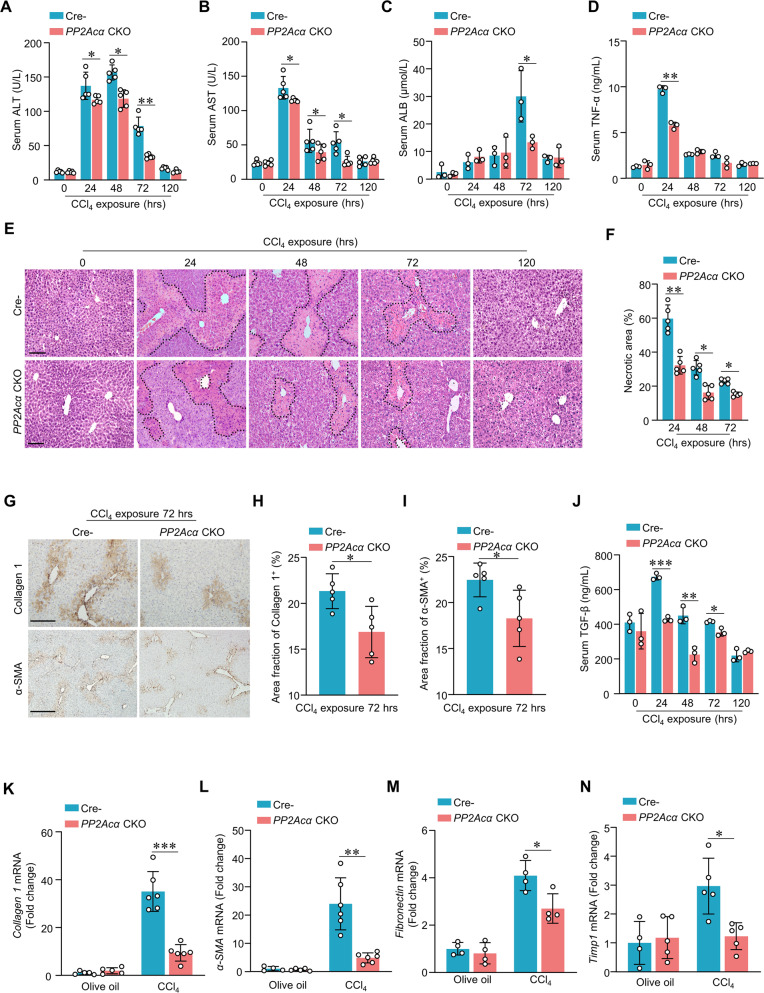
Deficiency of PP2Acα in hepatocytes alleviated ALI. Serum ALT (**A**) (*n* = 5), AST (B) (*n* = 5), total bile acid (**C**) (*n* = 3), and TNF-α levels (**D**) (*n* = 3) were analyzed in CCl_4_-induced ALI model mice. (**E** and **F**) Representative images of H&E staining of liver (**E**) (scale bar, 100 mm) and quantification of liver necrotic areas (**F**) in Cre^−^ and *PP2Acα* CKO mice treated with CCl_4_ from 0 to 120 h (*n* = 5). Representative images of IHC staining of liver Collagen I and α-SMA (**G**) (scale bar, 100 mm) and quantification of liver Collagen I-positive area (**H**) (*n* = 5) and α-SMA-positive area (**I**) (*n* = 5) in Cre^−^ and *PP2Acα* CKO mice treated with CCl_4_ 72 h. Serum TGF-β levels were analyzed in CCl_4_-induced ALI model mice (*n* = 3). qRT-PCR of relative fibrogenesis marker, *Collagen I* (**K**), *α-SMA* (**L**), *Fibronectin* (**M**), and *Timp1* (**N**) mRNA levels in Cre^−^ and *PP2Acα* CKO mice treated with CCl_4_ or olive oil. *p < 0.05, **p < 0.01, ***p < 0.001, one-way ANOVA followed by Tukey’s multiple comparisons test (**A**–**D**, **F**, **J**–**N**); two-tailed Student’s unpaired *t* test (**H** and **I**). Data are represented as mean ± SD.

To further explore what role does PP2Acα play in CCl_4_-induced ALI, we compared liver injuries in *PP2Acα* cKO and Cre^−^ mice. Confirming successful model induction, the serum levels of alanine aminotransferase (ALT), aspartate aminotransferase (AST), and total bile acid—all of which are indicators of disrupted liver function—were all significantly increased upon CCl_4_ injection. Compared to Cre^−^ mice, the *PP2Acα* cKO ALI model mice had significantly reduced ALT, AST, and total bile acid levels (Fig. 5A–C). We also found that the TNF-α level was about 30% lower in *PP2Acα* cKO mice compared to wild-type controls at 24 h after CCl_4_ injection (Fig. 5D). H&E staining indicated that livers from *PP2Acα* cKO mice had significantly smaller necrotic areas from 24 h to 72 h after CCl_4_ injection compared to Cre^−^ control, just 24 h after CCl_4_ administration, the necrotic area in PP2Acα cKO mice was only 46% the size of the necrotic area of Cre^−^ mice (Fig. 5E, F). We also assessed the extent fibrosis in the ALI model mice. Compared to Cre^−^ ALI model livers, the *PP2Acα* cKO livers had lower levels of Collage I and α-SMA expression (Fig. 5G–I). The fibrogenesis levels and serum TGF-β levels were significantly lower in CCl_4_-treated *PP2Acα* cKO mice in vivo compared to Cre^−^ mice (Fig. 5J). Consistently, the mRNA levels of fibrogenesis-related genes such as *α-SMA*, *Collage I*, *Fibronectin*, *Tmp1*, *Tgf-β*, and *Pdgft-β* were down-regulated in *PP2Acα* cKO ALI model mice (Fig. 5K–N, Fig. S5A, B) Thus, it is clear that *PP2Acα* deficiency in hepatocytes reduces the extent of liver damage in ALI model mice.

### Inhibiting apoptosis with z-vad-fmk exacerbates liver damage in PP2Acα deficient ALI model mice

**Fig. 6:**
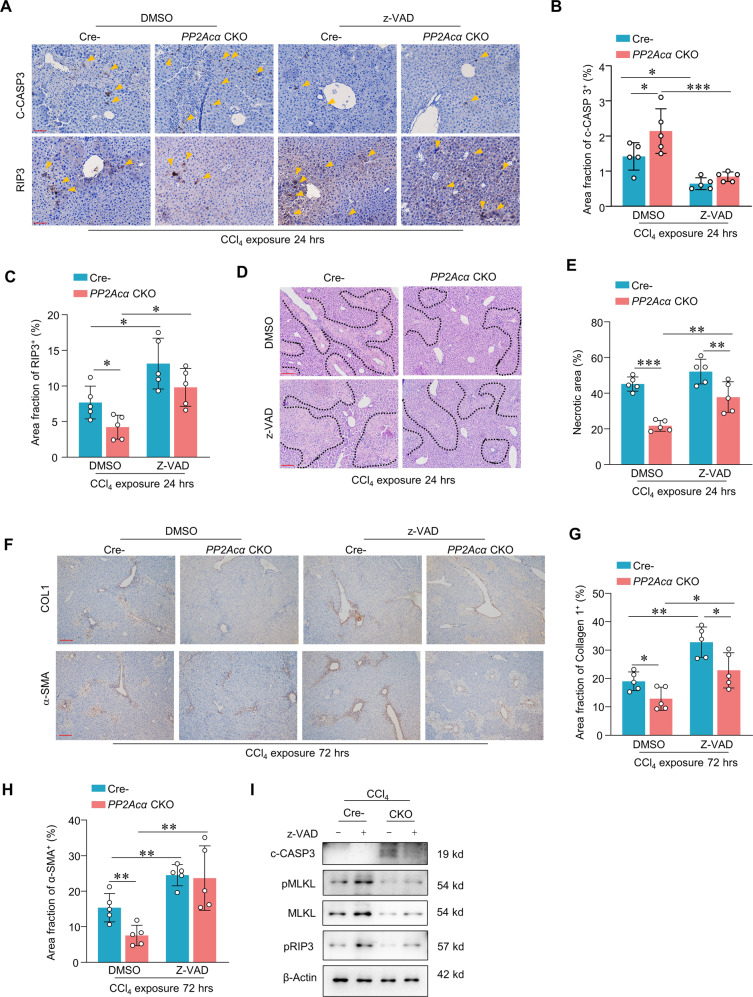
Inhibiting apoptosis by z-vad-fmk increased the damage in ALI. Representative images of IHC staining of liver c-CASP3 and RIP3 (**A**) (scale bar, 100 mm) and quantification of liver c-CASP3-positive area (**B**) and RIP3-positive area (**C**) in Cre^−^ and *PP2Acα* CKO mice treated with z-vad-fmk 0.5 h, followed by CCl_4_ 24 h treatment (*n* = 5) Representative images of H&E staining of liver (**D**) (scale bar, 200 mm) and quantification of liver necrotic areas (**E**) in mice treated as indicated (*n* = 5). Representative images of IHC staining of liver Collagen I and α-SMA (**F**) (scale bar, 200 mm) and quantification of liver Collagen I-positive area (**G**) and α-SMA-positive area (**H**) in mice treated as indicated (*n* = 5). **I** Immunoblotting showed the liver c-CASP3, pRIP3, pMLKL, and MLKL in Cre^−^ or *PP2Acα* CKO mice treated with z-vad-fmk 0.5 h, followed by CCl_4_ 24 h treatment.*p < 0.05, **p < 0.01, ***p < 0.001, one-way ANOVA followed by Tukey’s multiple comparisons test (**B**, **C**, **E**, **G** and **H**). Data are represented as mean ± SD.

Recall our findings that SMAC Mimetic SM-164 leads to smaller necrotic areas and lower AST and ALT levels in CCl_4_-induced ALI mice (Fig. 2), which together suggest it maybe possibly alleviate ALI by somehow biasing the preference of hepatocytes towards apoptosis over necroptosis. To explore whether *PP2Acα* deficiency alleviates ALI by preferencing apoptosis over necroptosis, we intraperitoneally injected *PP2Acα* cKO mice and Cre^−^ mice with the known apoptosis inhibitor z-Vad-fmk 30 min prior to the CCl_4_ ALI model induction. Confirming that the inhibitor did reduce the extent of apoptosis, immunostaining revealed that liver sections from Cre^−^ or *PP2Acα* cKO mice at 24 h showed a 50% reduction in the signals for c-CASP3 (Fig. 6A, B). The z-Vad-fmk treatment induced RIP3 accumulation and increased the size of the necrotic area (Fig. 6A, C–E), significantly increased the α-SMA and collagen I positive areas in Cre^−^ and *PP2Acα* cKO mice (Fig. 6F–H). Immunoblotting assay showed z-Vad-fmk shifting apoptosis to necroptosis by upregulation of necroptosis-related protein (pRIP3 and pMLKL), and down-regulation of c-CASP3 in Cre^−^ mice (Fig. 6I). These results showing that experimentally limits apoptosis in *PP2Acα* deficient ALI model mice exacerbates liver damage and increases necroptosis.

### PP2Acα deficiency alleviated ALI through ASK/JNK pathway

**Fig. 7:**
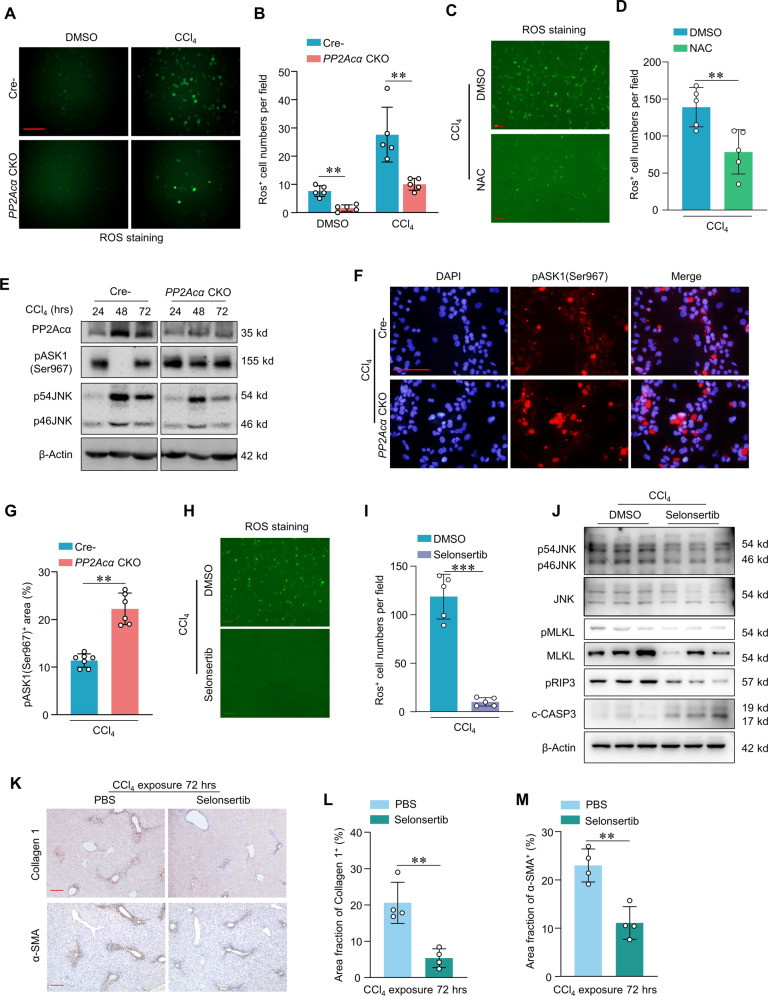
PP2Acα was involved in apoptosis and necroptosis via ASK/JNK signaling. Representative images (**A**) (scale bar, 100 μm) and quantification of ROS staining (**B**) (*n* = 5) of primary hepatocytes isolated from Cre^−^ and *PP2Acα* CKO mice with DMSO or CCl_4_ treatment. Representative images (**C**) (scale bar, 50 μm) and quantification of ROS staining (**D**) (*n* = 5) of primary hepatocytes stimulated with 5 nM NAC + CCl_4_ or DMSO + CCl_4_ administration for 24 h. **E** Immunoblotting showed the liver PP2Acα, p-ASK1(Ser967), p54JNK, p46JNK, β-Actin in Cre^−^ and *PP2Acα* CKO mice with CCl_4_ induction 24, 48, and 72 h. Representative IF images (**F**) (scale bar, 100 μm) and quantification of p-ASK1(Ser967) staining (**G**) (*n* = 6, 7) in primary Cre^−^ and *PP2Acα* CKO hepatocytes treated with CCl_4_. Representative images (**H**) (scale bar, 50 μm) and quantification of ROS staining (**I**) (*n* = 5) of primary hepatocytes stimulated with 10 nM ASK inhibitor selonsertib or DMSO for 3 h and then received CCl_4_ administration. **J** Immunoblotting showed the pro-CASP3, c-CASP3, pRIP3, pMLKL, MLKL, p54JNK and p46JNK in primary hepatocytes with CCl_4_ + DMSO or CCl_4_ + selonsertib induction. Representative images of IHC staining of liver Collagen I and α-SMA (**K**) (scale bar, 200 mm) and quantification of liver Collagen I-positive area (**L**) (*n* = 4) and α-SMA-positive area (**M**) (*n* = 4) in WT mice treated with CCl_4_ 72 h, followed by Selonsertib or PBS treatment. *p < 0.05, **p < 0.01, ***p < 0.001, one-way ANOVA followed by Tukey’s multiple comparisons test (B); two-tailed Student’s unpaired *t*-test (**D**, **G**, **I**, **L** and **M**). Data are represented as mean ± SD.

Our preliminary data showed that oxidation-reduction processes were activated in CCl_4_-induced ALI by LC-MS/MS (Fig. 3B). Previous studies have indicated that JNK-dependent induction of reactive oxygen species (ROS) contributes to necroptosis induction [15] and have shown that PP2A can regulate TNF-α expression downstream of ASK1/JNK signaling [16]. We found that administrating CCl_4_ to cultured primary hepatocytes from *PP2Acα* cKO mice caused a significant decrease in ROS levels (Fig. 7A, B). And ROS inhibitor, NAC administration decreased the number of dead hepatocytes with CCl_4_ treatment (Fig. 7C, D). Additionally, we detected a sustained increase in the JNK protein level in control ALI model mice (Fig. 7E).

The dephosphorylation of ASK1 at Ser967 has been reported to activate ASK1, and ASK1 can subsequently phosphorylate and thereby activate JNK signaling [16]. Immunoblotting with antibodies against JNK and the phosphorylated form of ASK1 residue Ser967 showed that, compared to Cre^−^ ALI model mice, the *PP2Acα* cKO ALI mice had increased pASK1(Ser967) levels but decreased JNK levels (Fig. 7E). Immunofluorescence of cultured primary hepatocytes supported that CCl_4_ administration caused an increase in the accumulation of pASK1(Ser967) (Fig. 7F, G).

Therefore, we used the known ASK1 inhibitor selonsertib to block the ASK/JNK signaling pathway in CCl_4_-treated cultured primary hepatocytes isolated from Cre^−^ mice. We found that selonsertib treatment decreased the ROS level in CCl_4_-treated primary hepatocytes (Fig. 7H, I). Notably, the selonsertib treatment also decreased pJNK1/2 and p54 JNK expression compared with that in DMSO treatment group (Fig. 7J). Additionally, the selonsertib treatment also caused an increase in c-CASP3 accumulation and caused a reduction in lower pRIP3, pMLKL and MLKL expression as detected by immunoblotting in hepatocytes treated with CCl_4_, which suggested more apoptosis in different deadly stimulation (Fig. 7J). We also assessed the extent fibrogenesis in the ALI model mice with selonsertib treatment. Compared to PBS treatment group, the the selonsertib treatment decreased level of Collage I and α-SMA expression (Fig. 7K–M). These results support the hypothesis that PP2A/ASK/JNK pathway is critical in regulating the ratio of apoptosis vs. necroptosis during ALI.

## Discussion

PCD including apoptosis and necroptosis is a common feature of many liver diseases, including toxin-induced liver diseases such as alcoholic and non-alcoholic steatohepatitis [2]. Our research showed that CCl_4_ treatment leads to accidental cell death like necrosis (different from necroptosis) and PCDs in hepatocytes, including RIP3-driven necroptosis and caspase-3-driven apoptosis. We found that inducing more apoptosis in ALI mice liver can decrease the necroptosis levels and reduce liver damage. Therefore, we put forward the idea that biasing the type of PCD towards apoptosis may reduce liver damage. We used LC-MS/MS to explore the biological changes during ALI and found that PP2Acα was a key regulator, which was overexpressed in liver tissues of both subacute hepatitis patients and ALI mice. By using *PP2Acα* cKO mice, we found that deficiency of PP2Acα can alleviate liver injury and fibrogenesis by biasing the preference of hepatocytes towards apoptosis over necroptosis. The deficiency of PP2Acα can reduce the ROS production and thus inhibit necroptosis and promote apoptosis through ASK/JNK pathway.

During hepatotoxicity-induced liver injury, apoptosis is a stress response to the injury and is typically regarded as harmful; apoptosis is therefore considered to be a useful indicator for the severity of liver damage [17, 18]. Previous work has demonstrated that inhibition of apoptosis in liver diseases can alleviate liver diseases such as non-alcoholic steatohepatitis [19, 20]. We found as expected that treatment with the SMAC Mimetic SM-164 increases the extent of apoptosis in mouse livers and cultured primary hepatocytes. However, in the context of our CCl_4_-induced ALI model mice—in which both apoptosis and necroptosis are occurring—we observed that the SM-164-triggered apoptosis induction actually resulted in an overall reduction in necrotic tissue area, lower ALT and AST levels, and alleviated fibrogenesis. This surprising finding suggested that preferencing apoptosis over necroptosis in ALI livers may help reduce the severity of ALI-related damage in livers. Apoptosis is widely studied and quite complex, and beyond simply responding to injury by causing locally damaged cells to die, apoptotic processes have been shown to protect the viability of surrounding cells. That is, cells undergoing apoptosis culminate with the release of apoptotic bodies which carry trigger “find-me” and “eat-me” signals that are recognized by phagocytotic cells. These phagocytotic cells thus take up the apoptotic bodies, a process which prevents excessive cellular leakage, thereby promoting a non-inflammatory (or low inflammatory) microenvironment [2, 21, 22]. In contrast, necroptotic cells have a morphology characterized by incomplete plasma membranes, which directly causes the leakage of pro-inflammatory damage associated molecular patterns (DAMPs); these DAMPs are then present in the microenvironment, where they activate innate immune responses that can further exacerbate liver injury [3, 23]. Although apoptosis and necroptosis are both forms of PCD, these stark differences in their respective cellular damage mechanisms help to support our surprising finding about the potential benefit of preferencing apoptosis over necroptosis to reduce the extent of liver injury.

Apoptosis and necroptosis do not occur in isolation and can shift between each other. Activation of caspase-8 may shift the balance away from necrosis and towards apoptosis by cleaving RIP1 and RIP3, whereas inhibition of caspase-8 leads to assembly of pro-necroptotic RIP1/RIP3 complexes [24, 25]. Our results indicated that PP2Acα performs a switching function between apoptosis and necroptosis in hepatocytes. PP2Acα’s switching function involves ROS and the ASK/JNK signaling pathway. A previous study of cultured kidney cells showed that ROS can activate ASK1 by causing PP2A-mediated dephosphorylation of pASK1 (Ser967) [26]. This ASK1 dephosphorylation promotes necroptosis by inducing sustained activation of JNK [16, 27]. Furthermore, the deletion of *PP2Acα* in hepatocytes caused a reduction in ROS levels, an increase in pASK1 (Ser967) accumulation, and a decreased extent of JNK activation, which together contributed to a substantial induction in necroptosis. Inhibition of ASK1 by selonsertib in hepatocytes could also change the preference of cell death program. Interestingly, blocking PP2Acα-ASK1 signaling caused reduction in both MLKL and pMLKL protein levels. A possible explanation for this might be that the transcription factors for regulation of *MLKL* gene transcription were controlled by PP2Acα-ASK1-JNK signaling. Further investigation is required to confirm this regulatory mechanism.

Our results establish that PP2Acα in CCl_4_-induced ALI. Knockout of *PP2Acα* biased PCD in hepatocytes towards increased apoptosis and reduced necroptosis. Importantly, we found that this *PP2Acα*-deficiency-induced shift in PCD preference alleviated overall liver necrosis and led to substantially improved liver function. And these improvements were accompanied by lower serum levels of inflammatory factors such as TNF-α and TGF-β1. Pursuing this further, we found that the treatment of *PP2Acα* deficient ALI model mice with the apoptosis inhibitor z-Vad-fmk exacerbated necrosis, supporting that PP2Acα modulates liver injury by biasing PCD in hepatocytes towards an apoptotic rather than necroptotic fate. This conclusion is consistent with a study reporting that PP2A drives RIP1-dependent necroptosis in lung cancer [28], and our results highlight that PP2A merits further exploration as an excellent candidate for inducible switching between two PCDs pathways known to be active in the pathogenesis of ALI.

Studies of ALI have revealed that non-parenchymal cells including HSCs engage in extensive crosstalk with hepatocytes. During ALI, HSCs are induced to generate fibrogenic extracellular matrix components like collagen I [29]. We found that *PP2Acα* deficiency limited fibrogenesis in ALI, and our inducer/inhibitor assays confirmed that elevated necroptosis in hepatocytes results in relatively more severe fibrosis phenotypes compared to elevated apoptosis. Therefore, it is plausible that therapeutically inhibiting PP2Acα should reduce liver fibrosis and alleviates liver injury by preferencing apoptosis over necroptosis.

In conclusion, our study demonstrates that deletion of *PP2Acα* in hepatocytes promotes apoptosis over necroptosis, which ultimately creates a favorable microenvironment that protects hepatocytes from ALI through ASK/JNK signaling. The improvements in liver tissue viability and liver function and reduced necrosis and fibrosis that we observed in the preferentially apoptotic ALI model mice were quite substantial, underscoring that further exploration of the therapeutic manipulation to preference particular forms of PCD may offer large benefits for patients of liver diseases like ALI.

## Materials and methods

### Human tissue samples

ALI tissues were obtained from 11 subacute hepatitis patients and 4 hemangioma adjacent tissues were used as healthy controls. All specimen collection procedures were approved by human research committee of Nanjing Drum Tower Hospital. Informed consent was obtained from each patient and the study was carried out according to the ethical guidelines of the 1975 Declaration of Helsinki.

### Animals

The hepatocyte-specific *PP2Acα* conditional knockout (cKO) mice used in this study were gifts from Prof. Xiang Gao (Model Animal Research Centre of Nanjing University) by breeding PP2Acα-floxed mice with albumin-Cre transgenic (Alb-Cre) mice [11]. Their littermates were used as control. Specific pathogen-free male mice from a mixed genetic background (129SV and C57/BL6) with aged 8–10 weeks were used for experiments. Mice were housed under standard conditions with a 12 h light-dark cycle and were fed with regular food and water ad libitum. All the mice were randomly group by a random-group-generator (https://www.randomready.com/random-group-generator/). And the experiments were done in a blinded fashion. All experimental procedures were reviewed and approved by the Animal Care Committee of Nanjing University in accordance with the Institutional Animal Care and Use Committee guidelines. No animals were excluded in this study.

### ALI model

The hepatocyte-specific *PP2Acα* conditional knockout (cKO) male mice were given an intraperitoneal (i.p.) injection with a 1:5 of CCl_4_ and olive oil at a dose of 1 μl/g body weight, and the control group only received olive oil (*n* ≥ 8 per experimental group). Tissues and serum were harvested at indicated time after CCl_4_ administration. SMAC Mimetic SM-164 was injected by caudal vein with 3 mg/kg body weight 30 min before CCl_4_ treatment. Apoptosis inhibitor z-Vad-fmk was intraperitoneally injected with 5 mg/kg body weight 30 min before CCl_4_ treatment.

Additional methods are described in the Supplementary Information-1.

## Supplementary information

Supplementary Information-1

Table S1

Table S2

Table S3

Table S4

Figure S1

Figure S2

Figure S3

Figure S4

Supplementary Information-2

aj-checklist

## Data Availability

All datasets generated and analyzed during this study are included in this published article and its Supplementary Information files (Unprocessed Western blots were provided in Supplementary Information-2). Additional data are available from the corresponding author on reasonable request.
