# Supplementary material for: Manipulating PP2Acα-ASK-JNK signaling to favor apoptotic over necroptotic hepatocyte fate reduces the extent of necrosis and fibrosis upon acute liver injury
Source: Cell Death Dis. 2022 Nov 22;13(11):985. doi: 10.1038/s41419-022-05353-z (PMC9684557; doi:10.1038/s41419-022-05353-z)
Supplement: Supplementary file 1 — Supplementary Information-1 [file 41419_2022_5353_MOESM1_ESM.docx]

Supplementary Information-1

Manipulating PP2Acα-ASK-JNK signaling to favor apoptotic over necroptotic hepatocyte fate alleviates acute liver injury

Ke Lu, Si-Yu Shen, Ou-Yang Luo, Yue Lu, Tian-Shu Shi, Jing Wu, Qi Cheng, Hua-Jian Teng, Di Chen, Xiang Lu, Chao-Jun Li, Qing Jiang, Lei Fang, Bin Xue.

**Materials and Methods**

*ALI model*

The hepatocyte-specific *PP2Acα* conditional knockout (cKO) male mice were given an intraperitoneal (i.p.) injection with a 1:5 of CCl_4_ and olive oil at a dose of 1 μl/g body weight, and the control group only received olive oil (n ≥ 8 per experimental group). Tissues and serum were harvested at indicated time after CCl_4_ administration. SMAC mimetic SM-164 was injected by caudal vein with 3 mg/kg body weight 30 minutes before CCl_4_ treatment. Apoptosis inhibitor z-Vad-fmk was intraperitoneally injected with 5 mg/kg body weight 30 minutes before CCl_4_ treatment.

*Chronic liver injury models*

*PP2Acα* cKO mice were given 1:5 of CCl_4_ and olive oil i.p. at a dose of 1 μl/g body weight every other day till 4 weeks after the first administration (n = 10 per experimental group). Smac mimetic SM-164 (A8815, APExBIO) were injected by caudal vein with 3 mg/kg weight 30 minutes before CCl_4_ treatment. Apoptosis inhibitor z-Vad-fmk (A1902, APExBIO) were intraperitoneally injected with 5 mg/kg weight 30 minutes before CCl_4_ treatment.

*LC-MS/MS*

Protein extraction, LC-MS/MS detection and data analysis were carried out as descripted [1].

*Histological analysis*

The liver samples were fixed in 4% PFA, dehydrated in graded concentration of ethanol and embedded in paraffin for histopathological analysis. At least three sections were chosen from each group and all the sections were deparaffinized and rehydrated before detection.

*Hematoxylin and eosin (H&E) staining*

Sections were stained with hematoxylin for 5 minutes followed by counterstaining with eosin for 30 seconds. The necrotic hepatocytes were stained lighter by eosin. The slightly stained areas of hepatocytes around the central vein were calculated by ImageJ software. At least 4 fields per section were selected and all graphs were captured by Olympus BX51 Fluorescence Microscope.

*Immunohistochemistry*

Sections were pretreated with 3% H_2_O_2_ for 10 minutes at room temperature followed by washing with dH_2_O. Then sections were submitted to antigen retrieval by incubation in citrate buffer (pH 6.0) steamed for 8 minutes and subsequently blocked-in normal goat serum for 1 hour. Next, the sections were incubated with primary antibodies against PP2Acα (1/400) (ab137825, Abcam, Cambridge, UK), phospho-(Ser/Thr) Phe (1/200) (ab17494, Abcam), PPP1CA (1/200) (A12468, ABclonal, Wuhan, China), PPP5C (1/200) (A8753, ABclonal), PPP6C (1/200) (A4039, ABclonal), cleaved caspase-3 (1/500) (#9661S, Cell Signaling Technology, Danvers, USA), RIP3 (1/400) (ab152130, Abcam), α-SMA (1/400) (sc-53142, Santa Cruz Biotechnology, Dallas, USA) or collagen I (1/400) (Santa Cruz Biotechnology) overnight at 4°C. The sections were then incubated with secondary antibody (1:300) (horseradish peroxidase-conjugated anti-rabbit or anti-mouse IgG) and developed by diaminobenzidine (DAB).

*Masson Staining*

For Masson trichrome staining, the sections were stained using Masson Staining Kit according to the manufacturer’s instructions (KGMST-8003, Jiangsu KeyGEN BioTECH, Nanjing, China). All of the 15 patients’ tissue samples were observed and at least 4 fields per sample were selected.

*TUNEL assay*

TUNEL assay was performed using the DeadEnd™ Fluorometric TUNEL System (G3250, Promega, Madison, USA) according to the manufacturer’s instruction. After dewaxing and rehydration, tissue sections were permeabilized with Proteinase K. Then the incubation buffer was added and incubated at 37°C for 1 hour avoiding light. After dipped in 2x SSC, slides were incubated with DAPI for 15 minutes and detected by fluorescence microscopy. The TUNEL positive cells were counted and at least 4 fields per sample were selected and graphs were captured by Olympus BX51 Fluorescence Microscope.

*Serum analysis*

Serum was isolated from blood as a supernatant fraction following centrifugation at 1500 rpm for 10 min. The activities of serum aspartate transaminase (AST), alanine transaminase (ALT) and total bile acid (TBA) were measured by fully automatic chemistry analyzer (Auto Chemistry, Chemray420, Rayto Life and Analytical Sciences, China). The quantities of TNF-α and TGF-β in liver serum or cell medium supernatant were determined using enzyme-linked immunosorbent assay kits (Boster, China; Cloudclone, USA) according to the manufacturer’s instructions.

*Immunocytochemistry and immunofluorescence*

Cells cultured on glass sheets were first fixed in 4% PFA for 30 min and washed with PBS. The cells were permeabilized by PBS containing 0.5% Triton-X-100 and 0.5% Tween 20 and blocked by goat serum for 1 hour at room temperature. Cells were incubated with anti-Collagen-I and anti-p-ASK1(Ser967) (#3764，Cell Signaling Technology) antibodies overnight at 4°C. For immunocytochemistry, the cells were incubated with corresponding secondary antibodies for 90 minutes and developed by diaminobenzidine (DAB). For immunofluorescence, the cells were incubated with rabbit IgG (H+L) Cross-Adsorbed Secondary Antibody in IF (1/300) (A-11012, Thermo Fisher Scientific, Waltham, USA) for 1 hour at room temperature in the dark. Counterstaining was performed using DAPI (1/600) for 10 min at room temperature. Results of immunofluorescence were detected by Olympus BX51 Fluorescence Microscope.

*Primary hepatocytes and hepatic stellate cells isolation and treatment*

Primary hepatocytes and hepatic stellate cells were isolated as described previously [2]. Briefly, after perfusion and digestion, the surrounding capsule of the liver was removed and dissociated in 10 mL 10% DMEM medium, and then filtered through 70 μm cell strainers and centrifuged for 5 min at 50× g at 4°C. The supernatant was transferred to a new centrifuge tube and centrifuged for 10 min at 2000× g at 4°C, then suspended the precipitate with 2 ml 10% FBS culture medium. A discontinuous density gradient (15%, 10%) was made using OptiPrepTM (Sigma-Aldrich, St. Louis, MO) following the manufacturer’s instructions and the cells were centrifuged for 10 min at 2000× g at 4°C. The HSCs at the interface were collected. The precipitate was suspended with 20 ml 40% percoll (Sigma-Aldrich, St. Louis, MO) plus solution, after gentle mix, the primary hepatocytes were centrifuged at 400× g for 10 min at 4°C. The primary hepatocytes were then treated with CCl_4_ (1 mM), TNF + SMAC Mimetic (TS) or TS + caspase inhibitor z-VAD-fmk (TSZ), respectively. ASK inhibitor selonsertib (10 nM, S8292 Selleckchem) was treated 3 hours before CCl_4_ treatment. Apoptosis was induced by 30 ng/ml TNF-α (50349-MNAE, Sino Biological) and 500 nM SM-164 (A8815, APExBIO) while necroptosis was induced by TNF-α (30 ng/mL), SM-164 (500 nM), and 25 μM z-Vad-fmk (A1902, APExBIO). Cells were harvest 24 hours after TS, or TSZ, or CCl_4_ treatment for following analysis.

*Cell Co-culture*

Primary hepatocytes were cultured in Roswell Park Memorial Institute-1640 medium plus 10% FBS and primary hepatic stellate cells were cultured in DMEM plus 10% FBS. For cell co-culture experiments, primary hepatocytes were first transfected with siPP2Acα or the negative control using GM siRNA-mate kit (GenePharma, Shanghai, China). The siPP2Acα sequence were shown in Table S3. After 48 hours of culture, Primary hepatocytes were treated with 2 mM CCl_4_ for 12 h. Then half of the medium was transferred into primary HSCs, which had been starved overnight. RNA, proteins and medium of primary HSCs were collected after the medium was transferred for another 24 hours for further analysis. The isolated primary cells were tested for mycoplasma free before they were used.

*Annexin V-FITC/PI flow cytometry analysis*

Cells were stained with Annexin V and PI by Annexin V-eGFP/PI Staining Apoptosis Kit following instructions offered by manufacturer (Jiangsu KeyGEN BioTECH). For flow cytometry analysis, the cells were washed, centrifuged, and resuspended in a suitable amount of stain buffer, and detected by BD FACSAria Flow Cytometer (BD Biosciences).

*Reactive oxygen species (ROS) detection*

Reactive Oxygen Species (ROS) Detection Kit was purchased from Jiangsu KeyGEN BioTECH. Primary hepatocytes from CKO and control mice were stimulated by CCl_4_, DMSO or NAC (5 nM, Mackin, N800425) and then ROS detection procedures were performed following the manufacturer’s instructions.

*Western blot*

Cells and liver tissue from patients or mice were lysed in RIPA buffer. Proteins were separated by 8~12% SDS-PAGE and transferred to a Polyvinylidene difluoride (PVDF) membrane. The membranes were blocked with goat serum, followed by incubation overnight at 4°C in antibodies against PP2Acα (1/800) (ab137825, Abcam), caspase-3 (1/1,000) (#9662S, Cell Signaling Technology), cleaved caspase-3 (1/1,000) (#9661S, Cell Signaling Technology), RIP3 (1/800) (ab152130, abcam), p-ASK ser967 (1/500) (#3764, Cell Signaling Technology), p-JNK(Thr183/Tyr185) (G9) (1/500) (#9255, Cell Signaling Technology), and pan-JNK (1/500) (#9252, Cell Signaling Technology), p-RIP3 (ab222320, Abcam) and p-MLKL (ab187091, Abcam). Then the membranes were incubated with corresponding secondary antibodies for 90 minutes. The films were exposed and the graph was captured by Tanon 6100 Chemiluminescent Imaging System (Tanon Science & Technology, Shanghai, China)

*Co-IP*

The Protein A/G Magnetic Beads were purchased from MedChemExpress (HY-K0202), and the process was followed by the manufacturer’s instruction. Generally, the magnetic beads were firstly washed and collected by magnetic stand, the RIPK3 antibody (17563-1-AP, Proteintech) and control rabbit IgG (AC500, Abclonal) were then bind to the magnetic beads. The target protein from the lysate were then immunoprecipitated and eluted. The final solution was immunoblotted by anti-RIPK3 (#15828, Cell signaling technology), anti-RIPK1(#3493, Cell signaling technology) and anti-MLKL (ab172868, Abcam). The Anti-Rabbit IgG, Light Chain Specific (SA00001-7L, Proteintech) was used as secondary antibody.

*RNA extraction and quantitative real-time polymerase chain reaction*

Total RNA was extracted from liver tissues and cells with Trizol according to the manufacturer’s protocol (Invitrogen, USA) and reverse transcribed with the ReverTra Ace kit (Toyobo, Japan). The cDNA was subjected to SYBR Green-based real-time PCR analysis on an Applied Biosystems 7300 Sequence Detection System. All quantification was performed in triplicates and normalized to an endogenous 18-strand RNA control. *PP2Acα*, *α-SMA*, *Collagen I*, *Ctgf*, *Pdgfrβ*, *Tgf-β1*, *Fibronectin* and *Timp1* expression were detected. The primer sequences were listed in Table S4.

*Statistical analysis*

The sample size for each experiment was determined based on our previous study [3]. All the data were expressed as mean ± standard deviation (S.D.). Statistical analyses were conducted using Prism GraphPad Prim 8.0 software. Unpaired Student’s *t-*test (for two groups) and one-way ANOVA (for multiple groups with one variable factor) were used followed by the Tukey-Kramer test. All experiments were performed and analyzed using 3 individual samples, unless otherwise mentioned. A *P*-value less than 0.05 was considered statistically significant. The experiments were randomized, and the investigators were blinded to allocation during experiments and outcome assessment.

**Reference**

1. Cao Y, Ding W, Zhang J, Gao Q, Yang H, Cao W*, et al.* Significant Down-Regulation of Urea Cycle Generates Clinically Relevant Proteomic Signature in Hepatocellular Carcinoma Patients with Macrovascular Invasion. *J Proteome Res* 2019, **18**(5)**:** 2032-2044.

2. Lai SS, Fu X, Cheng Q, Yu ZH, Jiang EZ, Zhao DD*, et al.* HSC-specific knockdown of GGPPS alleviated CCl4-induced chronic liver fibrosis through mediating RhoA/Rock pathway. *Am J Transl Res* 2019, **11**(4)**:** 2382-2392.

3. Lu K, Shi TS, Shen SY, Shi Y, Gao HL, Wu J*, et al.* Defects in a liver-bone axis contribute to hepatic osteodystrophy disease progression. *Cell Metab* 2022, **34**(3)**:** 441-457 e447.

**Supplemental Figure Legends**

**Supplemental Figure S1 related to Figure 2**

**Supplemental Figure S1. SMAC mimetic treatment in wild-type mice.**

(**A**) Representative images of H&E staining of liver (scale bar, 100 mm) in WT mice treated with SMAC mimetic or PBS for 24hrs. (**B** and **C**) Serum ALT (B) and AST (C) levels in WT mice treated with SMAC mimetic or PBS for 24hrs. (n = 5). * *p* < 0.05, ** *p* < 0.01, *** *p* < 0.001, two-tailed Student’s unpaired t test (B and C). Data are represented as mean ± SD.

**Supplemental Figure S2 related to Figure 3**

**Supplemental Figure S2.** **LC-MS/MS based proteomic profiling.**

(**A** and **B**) Compared with control mice treated with olive oil, the levels of proteins associated with down-regulated molecular functions(A) and up-regulated molecular functions (B) in CCl_4_-induced ALI was shown.

**Supplemental Figure S3 related to Figure 4**

**Supplemental Figure S3. Characterize efficiency of** **PP2Acα liver specific knockout**

(**A**) Immunoblotting showed the liver PP2Acα expression in Cre^-^ and *PP2Acα* CKO mice (n = 2). (**B**) qRT-PCR of *PP2Acα* mRNA expression in Cre^-^ and *PP2Acα* CKO mice (n = 9). (**C**) Ratio of PI^+^ cells vs Annexin V^+^ cells in primary Cre^-^ or *PP2Acα* CKO hepatocytes with PBS or TS or TSZ or CCl_4_ treatment (n = 3). * *p* < 0.05, ** *p* < 0.01, *** *p* < 0.001, two-tailed Student’s unpaired t test (B and C). Data are represented as mean ± SD.

**Supplemental Figure S4 related to Figure 5**

**Supplemental Figure S4. qRT-PCR analysis of fibrogenesis markers.**

(**A-B**) qRT-PCR of *Tgf-β* (A) and *Pdgfr-β* (B) mRNA levels in Cre^-^ and *PP2Acα* CKO mice treated with CCl_4_ or olive oil. * *p* < 0.05, one-way ANOVA followed by Tukey’s multiple comparisons test (A and B). Data are represented as mean ± SD.
