# Supplementary material for: Manipulating PP2Acα-ASK-JNK signaling to favor apoptotic over necroptotic hepatocyte fate reduces the extent of necrosis and fibrosis upon acute liver injury
Source: Cell Death Dis. 2022 Nov 22;13(11):985. doi: 10.1038/s41419-022-05353-z (PMC9684557; doi:10.1038/s41419-022-05353-z)
Supplement: Supplementary file 2 — Table S1 [file 41419_2022_5353_MOESM2_ESM.docx]

**Table S1. Information of subacute hepatitis and hemangioma patients.**

| **Patient information** | | | **Hemangioma** | **Subacute hepatitis** |
| --- | --- | --- | --- | --- |
| **Gender** | | | Male (N=4) | Male (n=11) |
| **Age** | | | 46.3 ± 3.1 | 48.3 ± 2.69 |
| **Liver function** | | ALT (U/L) | 33.1 ± 7.3 | 127.4 ± 52.5 |
|  |  | AST (U/L) | 21.9 ± 3.0 | 97.7 ± 31.8 |
|  |  | TBIL (umol) | 15.1 ± 2.4 | 64.6 ± 31.3 |
|  |  | ALB (g/L) | 41.9 ± 1.2 | 37.7 ± 3.0 |
| **Pathology** | Inflammation grade (G) | G=0~1 | 4 | 0 |
|  |  | G≥2 | 0 | 11 |
|  | Fibrosis stage (S) | S=0~1 | 3 | 0 |
|  |  | S≥2 | 1 | 11 |
| **Child-pugh score** | | | ­/ | 9.8 ± 1.3 |
