# Supplementary material for: Manipulating PP2Acα-ASK-JNK signaling to favor apoptotic over necroptotic hepatocyte fate reduces the extent of necrosis and fibrosis upon acute liver injury
Source: Cell Death Dis. 2022 Nov 22;13(11):985. doi: 10.1038/s41419-022-05353-z (PMC9684557; doi:10.1038/s41419-022-05353-z)
Supplement: Supplementary file 3 — Table S2 [file 41419_2022_5353_MOESM3_ESM.docx]

**Table S2. Apoptosis related proteins in ALI mice.**

| Accession | Name | CCl_4_ : Olive oil | Peptides≥5 |
| --- | --- | --- | --- |
| Q9D0M5 | Dynein light chain 2, cytoplasmic | 2.630487919 | 9 |
| Q61335 | B-cell receptor-associated protein 31 | 1.844582081 | 5 |
| O70310 | Glycylpeptide N-tetradecanoyltransferase 1 | 1.41559577 | 9 |
| P68510 | 14-3-3 protein eta | 1.38085413 | 17 |
| P62259 | 14-3-3 protein epsilon | 1.31409812 | 33 |
| P11862 | Growth arrest-specific protein 2 | 1.27473855 | 5 |
| P58281 | Dynamin-like 120 kDa protein, mitochondrial | 1.213703156 | 37 |
| P63101 | 14-3-3 protein zeta/delta | 1.204949498 | 21 |
| Q62418 | Drebrin-like protein | 1.187568784 | 14 |
| Q9CQV8 | 14-3-3 protein beta/alpha | 1.178339005 | 20 |
| P61982 | 14-3-3 protein gamma | 1.091513872 | 19 |
| P70335 | Rho-associated protein kinase 1 | 1.083773851 | 7 |
| P62897 | Cytochrome c, somatic | 1.042205572 | 27 |
| Q9QXS1 | Plectin | 1.012212396 | 12 |
| Q8K1M6 | Dynamin-1-like protein | 0.990421414 | 20 |
| P70444 | BH3-interacting domain death agonist | 0.98734194 | 5 |
| P70168 | Importin subunit beta-1 | 0.963313937 | 11 |
| P48678 | Prelamin-A/C | 0.896223426 | 19 |
| P13020 | Gelsolin | 0.845254123 | 9 |
| Q07813 | Apoptosis regulator BAX | 0.843375385 | 8 |
| Q02248 | Catenin beta-1 | 0.837157369 | 8 |
| P16546 | Spectrin alpha chain, non-erythrocytic 1 | 0.833160102 | 68 |
| O55111 | Desmoglein-2 | 0.829319119 | 5 |
| P14733 | Lamin-B1 | 0.800769329 | 9 |
| P68254 | 14-3-3 protein theta | 0.682841063 | 16 |
| P20152 | Vimentin | 0.591445446 | 23 |
