# Supplementary material for: Manipulating PP2Acα-ASK-JNK signaling to favor apoptotic over necroptotic hepatocyte fate reduces the extent of necrosis and fibrosis upon acute liver injury
Source: Cell Death Dis. 2022 Nov 22;13(11):985. doi: 10.1038/s41419-022-05353-z (PMC9684557; doi:10.1038/s41419-022-05353-z)
Supplement: Supplementary file 4 — Table S3 [file 41419_2022_5353_MOESM4_ESM.docx]

**Table S3. Sequence of siPP2Acα.**

| siRNA | Sequence(5’-3’) |
| --- | --- |
| PP2Acα(human) | GCAGACAGAUCACACAAGUTT |
|  | ACUUGUGUGAUCUGUCUGCTT |
| Scramble | UUCUCCGAACGUGUCACGUTT |
|  | ACGUGACACGUUCGGAGAATT |
