# Supplementary material for: Manipulating PP2Acα-ASK-JNK signaling to favor apoptotic over necroptotic hepatocyte fate reduces the extent of necrosis and fibrosis upon acute liver injury
Source: Cell Death Dis. 2022 Nov 22;13(11):985. doi: 10.1038/s41419-022-05353-z (PMC9684557; doi:10.1038/s41419-022-05353-z)
Supplement: Supplementary file 5 — Table S4 [file 41419_2022_5353_MOESM5_ESM.docx]

**Table S4. Primer sequences used.**

| Primer | | Forward (5’-3’) | Reverse (5’-3’) |
| --- | --- | --- | --- |
| Mus musculus | |  |  |
|  | PP2Acα | GAACAATAGCCAGTTATTCAGG | TAATGAGCAATGGTAAGGAGC |
|  | Col1a1 | GCTCCTCTTAGGGGCCACT | CCACGTCTCACCATTGGGG |
|  | α-SMA | GTCCCAGACATCAGGGAGTAA | TCGGATACTTCAGCGTCAGGA |
|  | TGFβ1 | TTGCCCTCTACAACCAACACAA | GGCTTGCGACCCACGTAGTA |
|  | Fibronectin | AAGACCATACCTGCCGAATG | GAACATGACCGATTTGGACC |
|  | Timp1 | CGAGACCACCTTATACCAGCG | ATGACTGGGGTGTAGGCGTA |
|  | 18S | GTCTGTGATGCCCTTAGATG | AGCTTATGACCCGCACTTAC |
|  | PDGFRb | AGGAGTGATACCAGCTTTAGTCC | CCGAGCAGGTCAGAACAAAGG |
| Homo sapiens | |  |  |
|  | α-SMA | TCTGGAGGCACAACTGGCATCGT | TACATATGTTGTCCCCCTGATAG |
|  | PP2Acα | CAAAAGAATCCAACGTGCAAGAG | CGTTCACGGTAACGAACCTT |
|  | TGFβ1 | GGCCAGATCCTGTCCAAGC | GTGGGTTTCCACCATTAGCAC |
|  | 18S | GTCTGTGATGCCCTTAGATG | AGCTTATGACCCGCACTTAC |
