# Supplementary figures and images for: Manipulating PP2Acα-ASK-JNK signaling to favor apoptotic over necroptotic hepatocyte fate reduces the extent of necrosis and fibrosis upon acute liver injury
Source: Cell Death Dis. 2022 Nov 22;13(11):985. doi: 10.1038/s41419-022-05353-z (PMC9684557; doi:10.1038/s41419-022-05353-z)

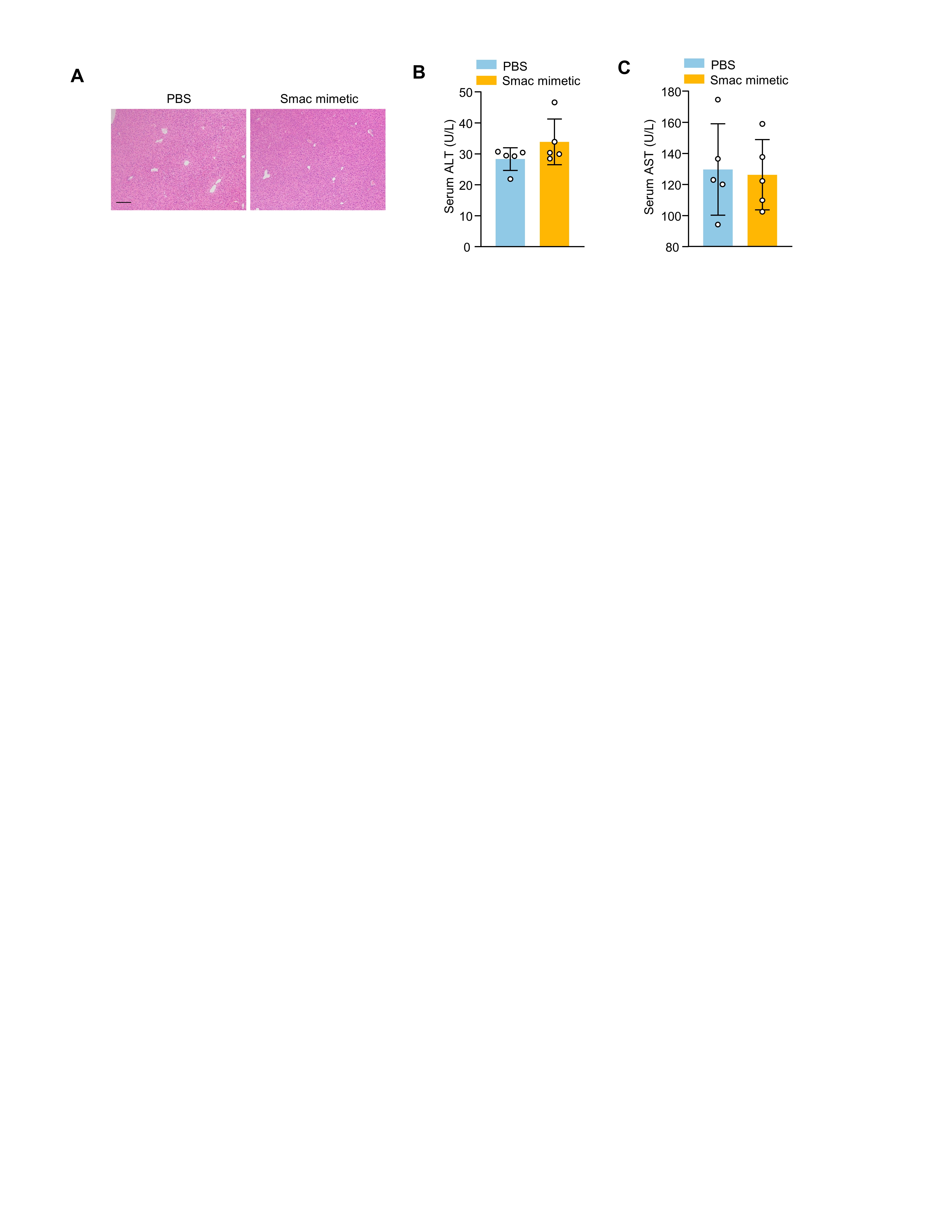

Supplement: Supplementary file 6 — Figure S1 [file 41419_2022_5353_MOESM6_ESM.jpg]

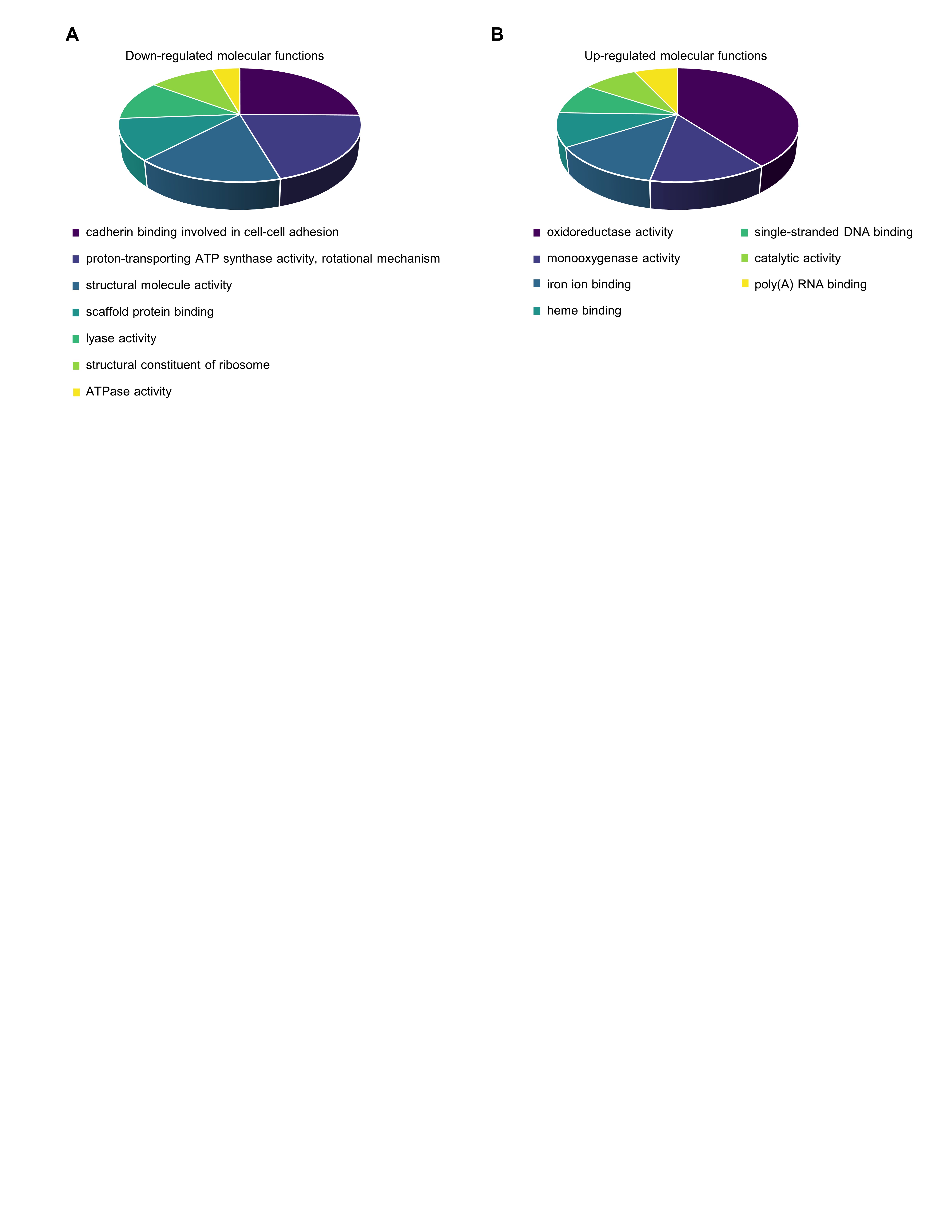

Supplement: Supplementary file 7 — Figure S2 [file 41419_2022_5353_MOESM7_ESM.jpg]

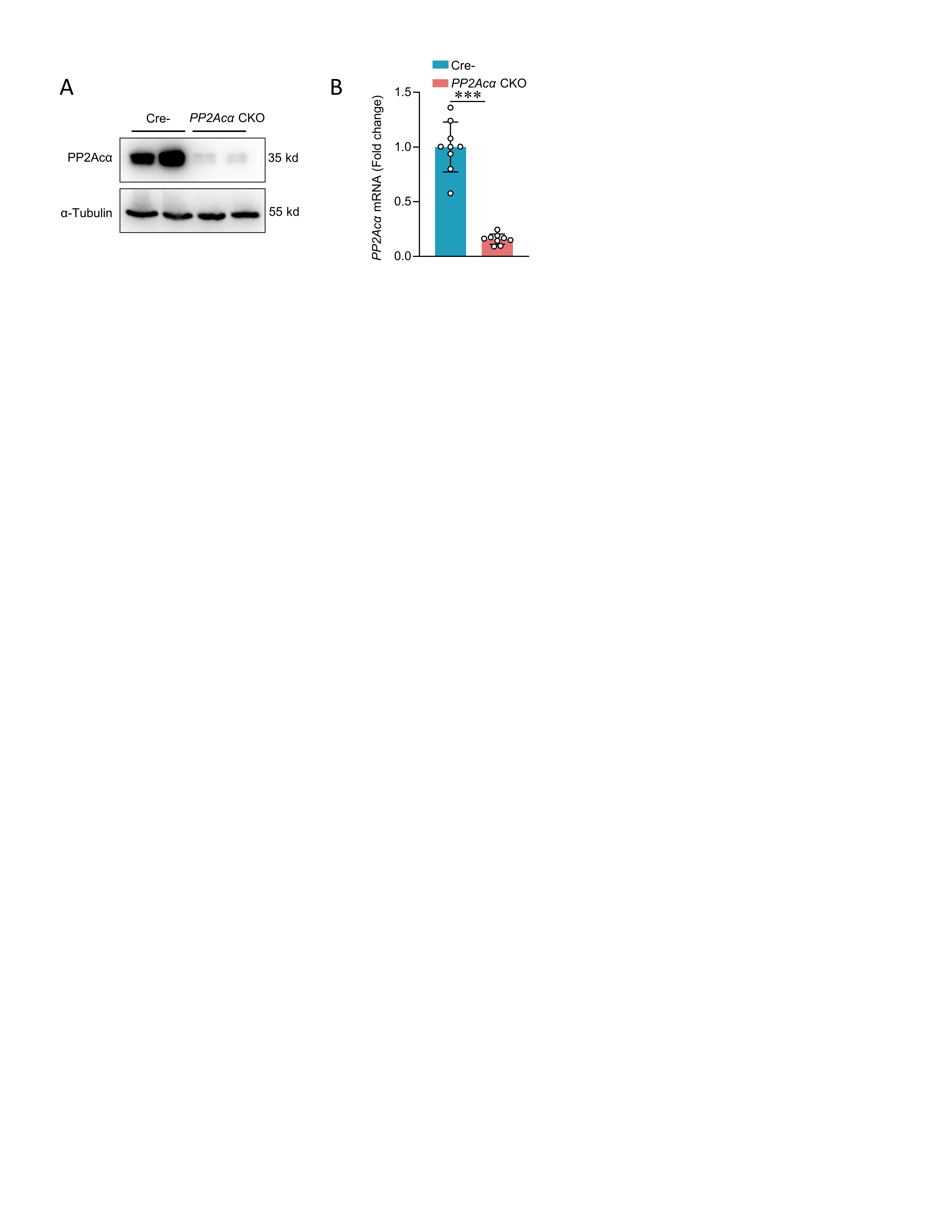

Supplement: Supplementary file 8 — Figure S3 [file 41419_2022_5353_MOESM8_ESM.jpg]

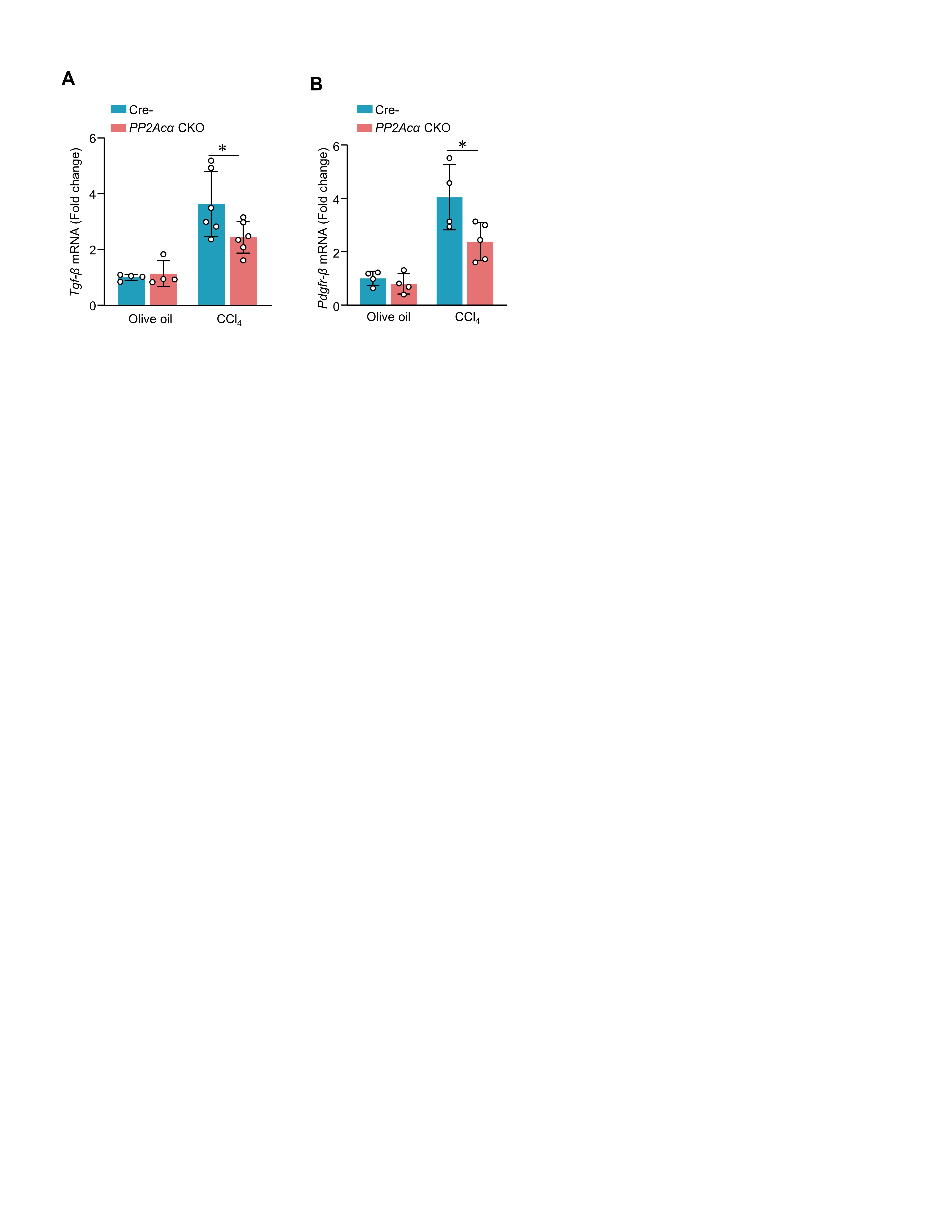

Supplement: Supplementary file 9 — Figure S4 [file 41419_2022_5353_MOESM9_ESM.jpg]
