# Supplementary material for: Manipulating PP2Acα-ASK-JNK signaling to favor apoptotic over necroptotic hepatocyte fate reduces the extent of necrosis and fibrosis upon acute liver injury
Source: Cell Death Dis. 2022 Nov 22;13(11):985. doi: 10.1038/s41419-022-05353-z (PMC9684557; doi:10.1038/s41419-022-05353-z)

Supplementary Information-2

Unprocessed Western blot

Figure 1H c-CASP3

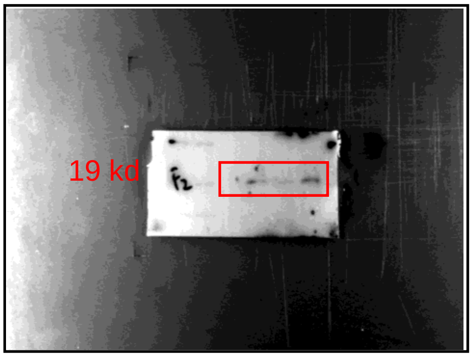

Figure 1H pMLKL

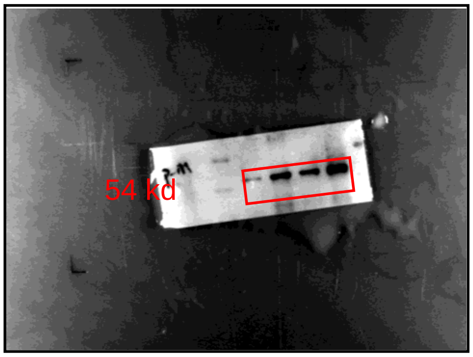

Figure 1H MLKL

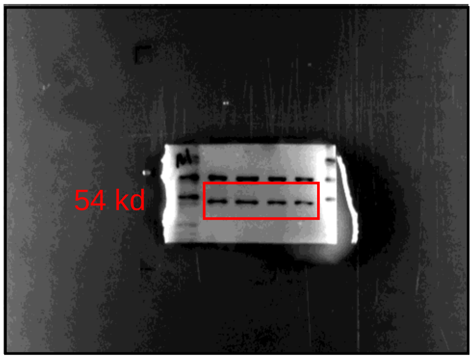

Figure 1H pRIP3

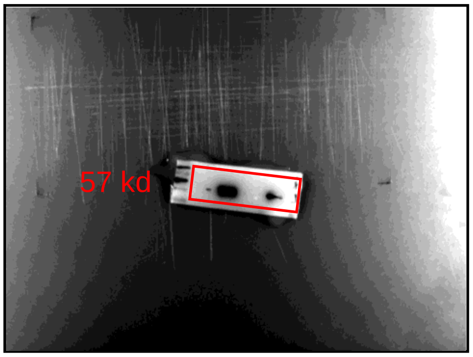

Figure 1H RIP3

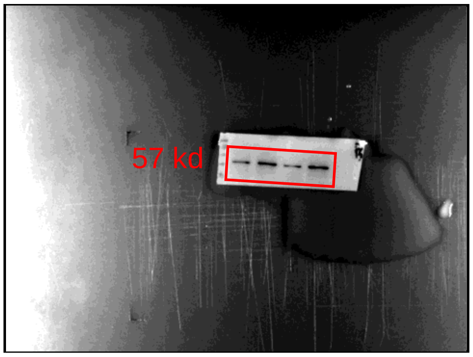

Figure 1H β-Actin

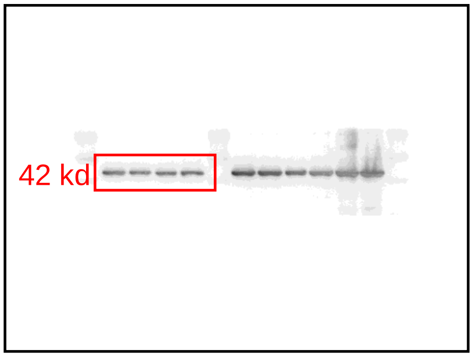

Figure 1I RIP3

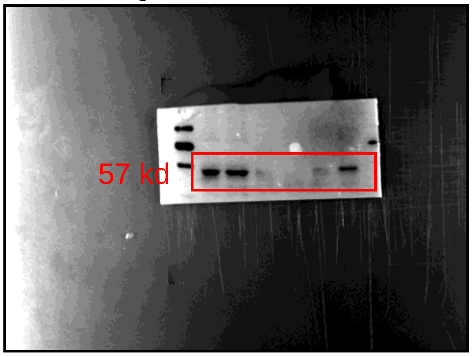

Figure 1I RIP1

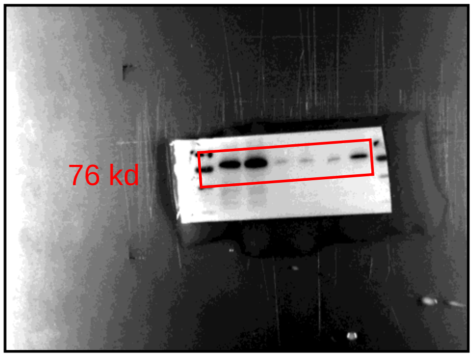

Figure 1I MLKL

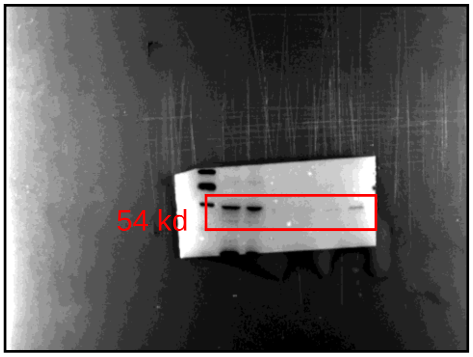

Figure 1I β-Actin

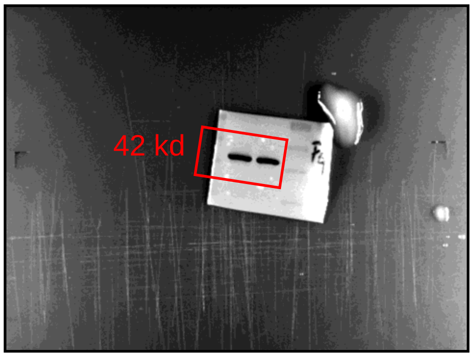

Figure 3E PP2Acα

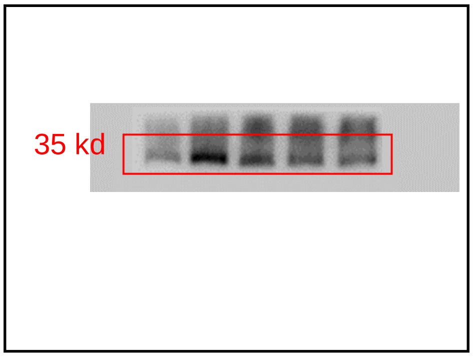

Figure 3E β-Actin

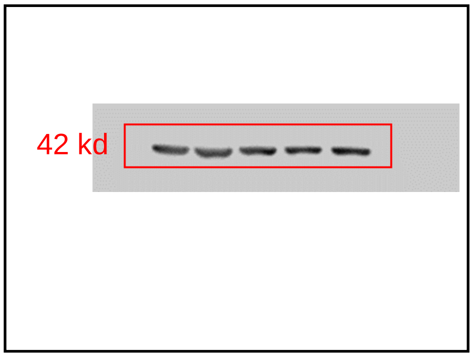

Figure 4C RIP3

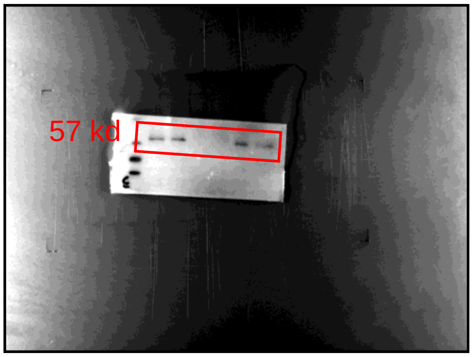

Figure 4C RIP1

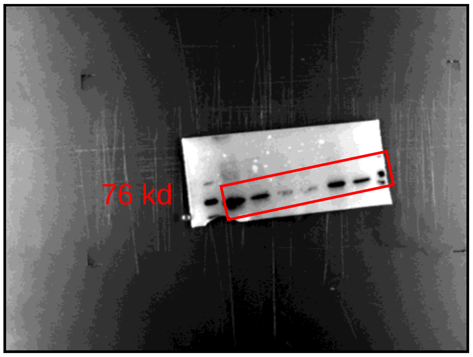

Figure 4C MLKL

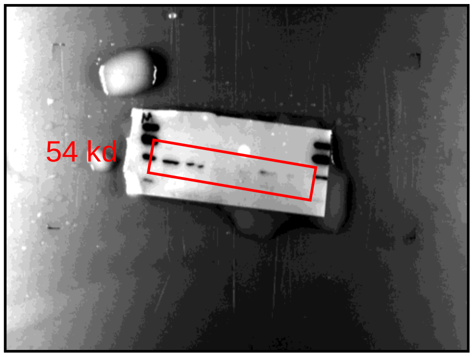

Figure 4C β-Actin

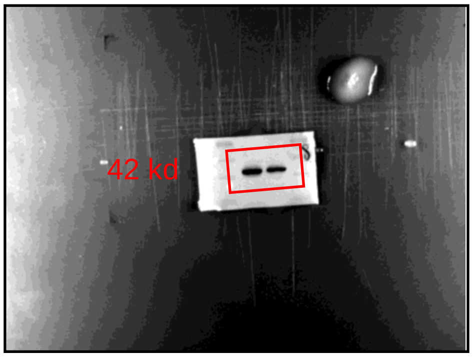

Figure 4I pro-CASP3, c-CASP3

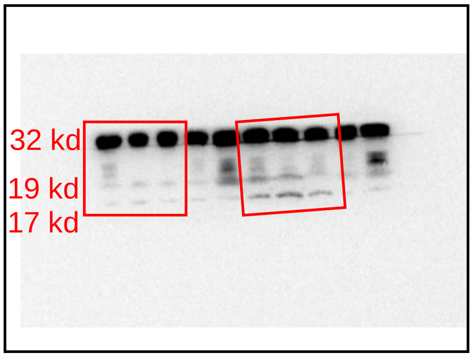

Figure 4I RIP3

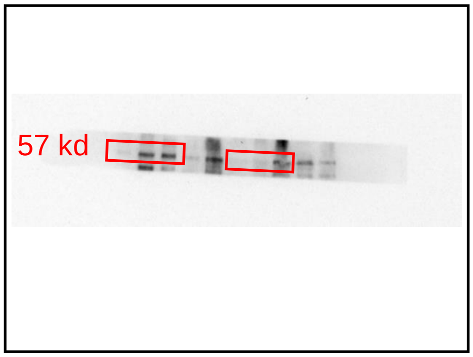

Figure 4I β-Actin

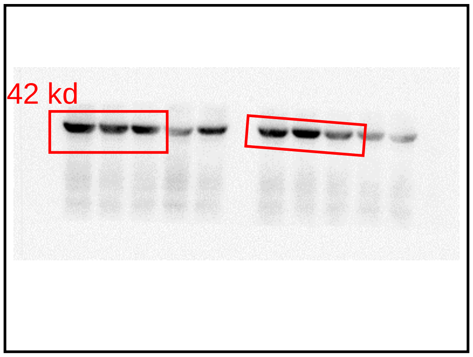

Figure 6I c-CASP3

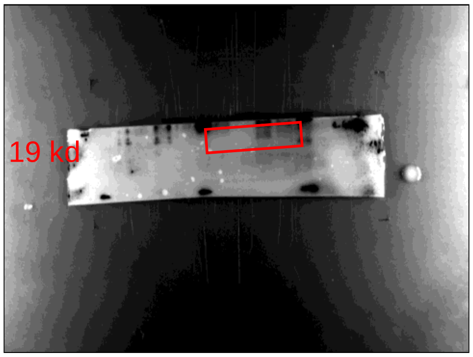

Figure 6I pMLKL

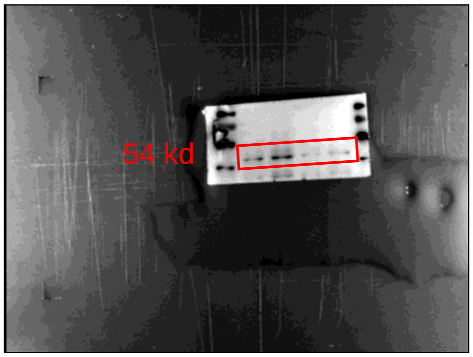

Figure 6I MLKL

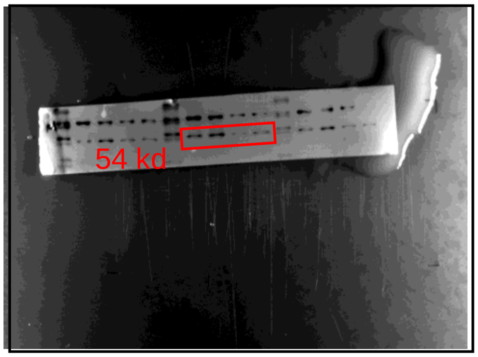

Figure 6I pRIP3

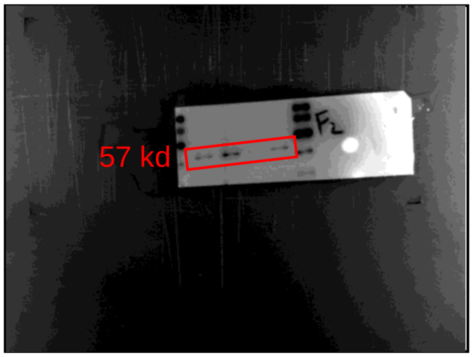

Figure 6I β-Actin

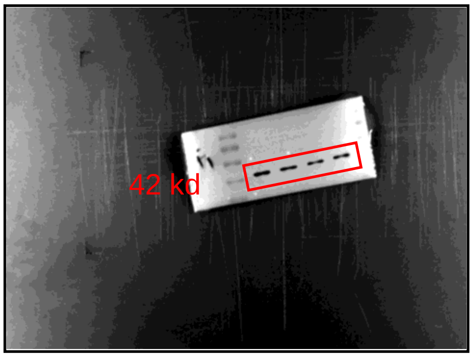

Figure 7E PP2Acα

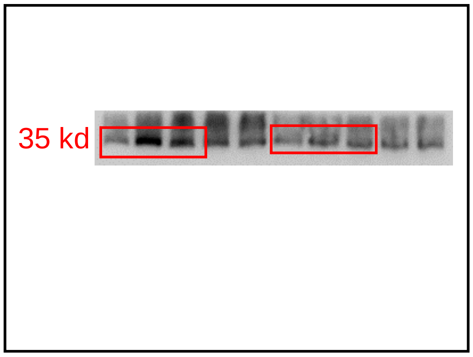

Figure 7E pASK1(Ser967)

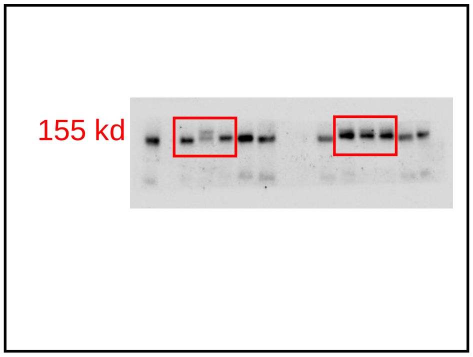

Figure 7E p54JNK, p46JNK

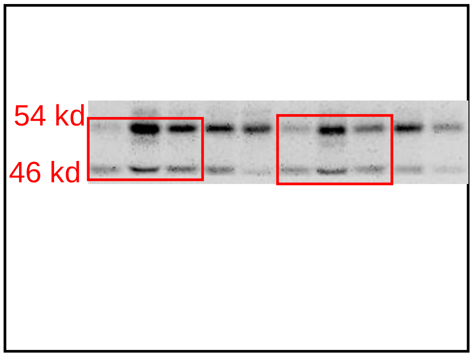

Figure 7E β-Actin

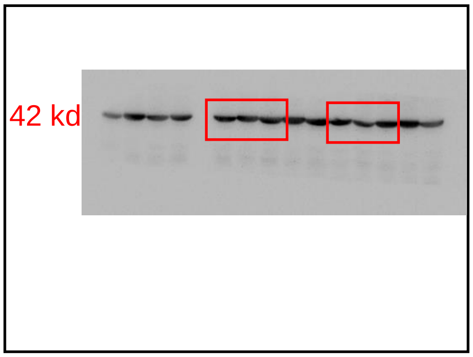

Figure 7J p54JNK, p46JNK

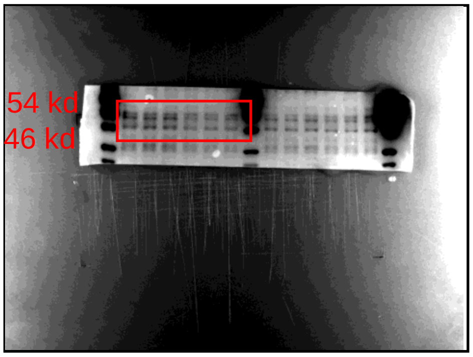

Figure 7J JNK

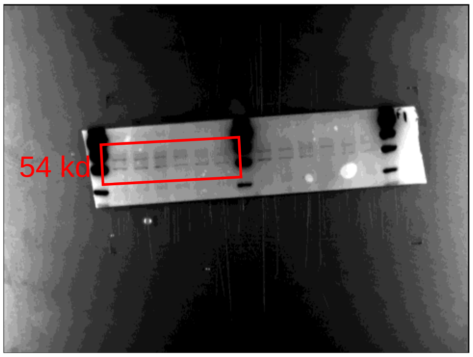

Figure 7J pMLKL

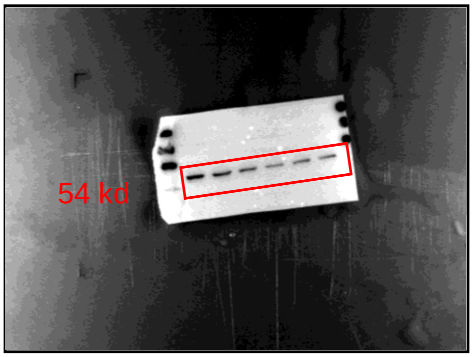

Figure 7J MLKL

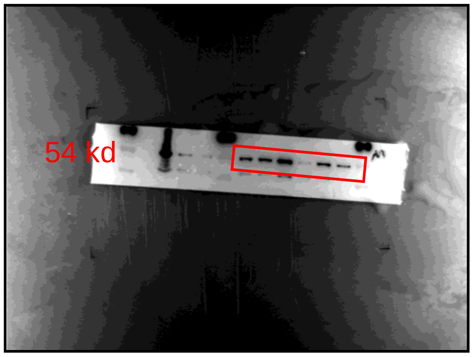

Figure 7J pRIP3

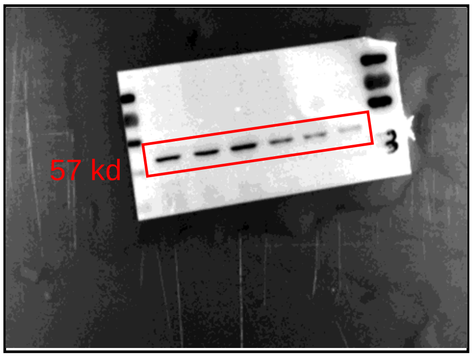

Figure 7J c-CASP3

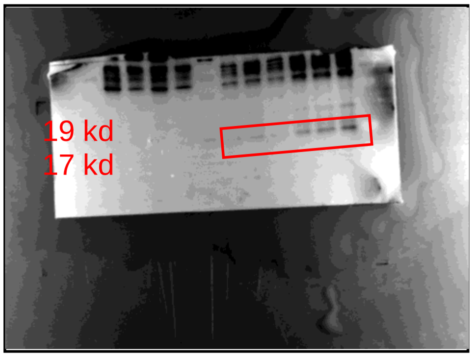

Figure 7J β-Actin

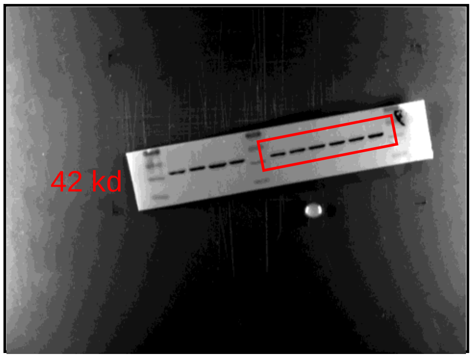

Figure S3A PP2Acα

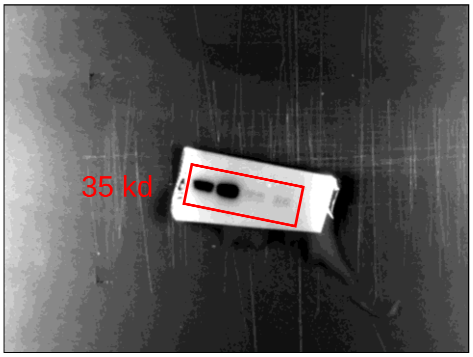

Figure S3A α-Tubulin

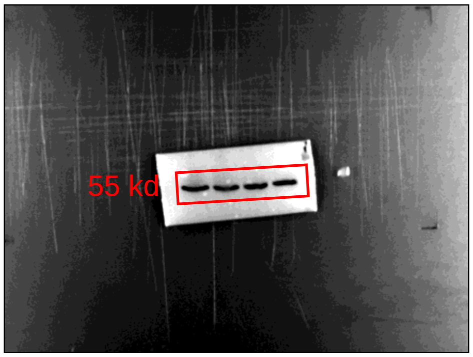

Supplement: Supplementary file 10 — Supplementary Information-2 [file 41419_2022_5353_MOESM10_ESM.pdf]
